# Supplementary material for: NADH Shuttling Couples Cytosolic Reductive Carboxylation of Glutamine with Glycolysis in Cells with Mitochondrial Dysfunction
Source: Mol Cell. 2018 Feb 15;69(4):581–593.e7. doi: 10.1016/j.molcel.2018.01.034 (PMC5823973; doi:10.1016/j.molcel.2018.01.034)
Supplement: Document S1. Figures S1–S7 [file mmc1.pdf]

Molecular Cell, Volume *69*

Supplemental Information

NADH Shuttling Couples Cytosolic Reductive Carboxylation of Glutamine with Glycolysis in Cells with Mitochondrial Dysfunction

Edoardo Gaude, Christina Schmidt, Payam A. Gammage, Aurelien Dugourd, Thomas Blacker, Sew Peak Chew, Julio Saez-Rodriguez, John S. O'Neill, Gyorgy Szabadkai, Michal Minczuk, and Christian Frezza

Supplementary Figure 1

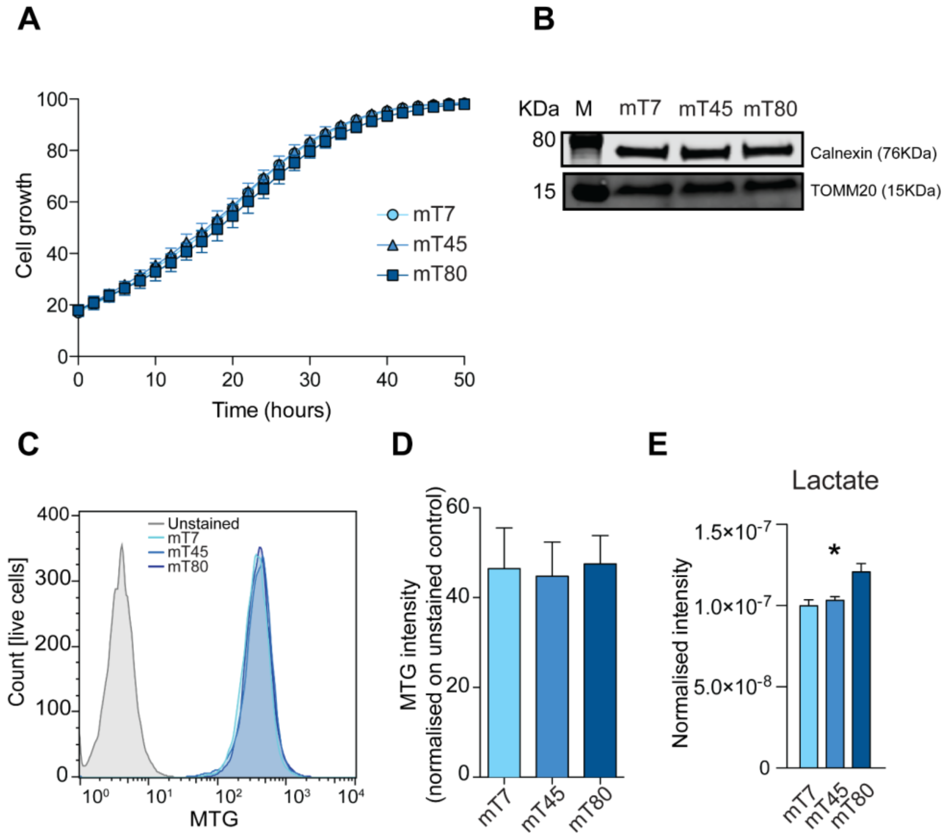

Supplementary Figure 1 related to Figure 1. Characterisation of cybrid cells. (A) Cell growth of mT7, mT45, and mT80 cells in standard conditions measured using Incucyte. (B) Western blot analysis of the mitochondrial membrane marker TOMM20. Calnexin was used as loading control. (C) Representative fluorescence distributions of cells with or without Mitotracker Green (MTG) staining from FACS analysis and (D) mean MTG intensity after subtraction of intensity from unstained control. Data are mean ± s.e.m. from three independent cultures. (E) Lactate secretion as measured in mT7, mT45 and mT80. All data are mean ± s.e.m. from four independent cultures. (E) * indicates ANOVA p-value < 0.05, respectively.

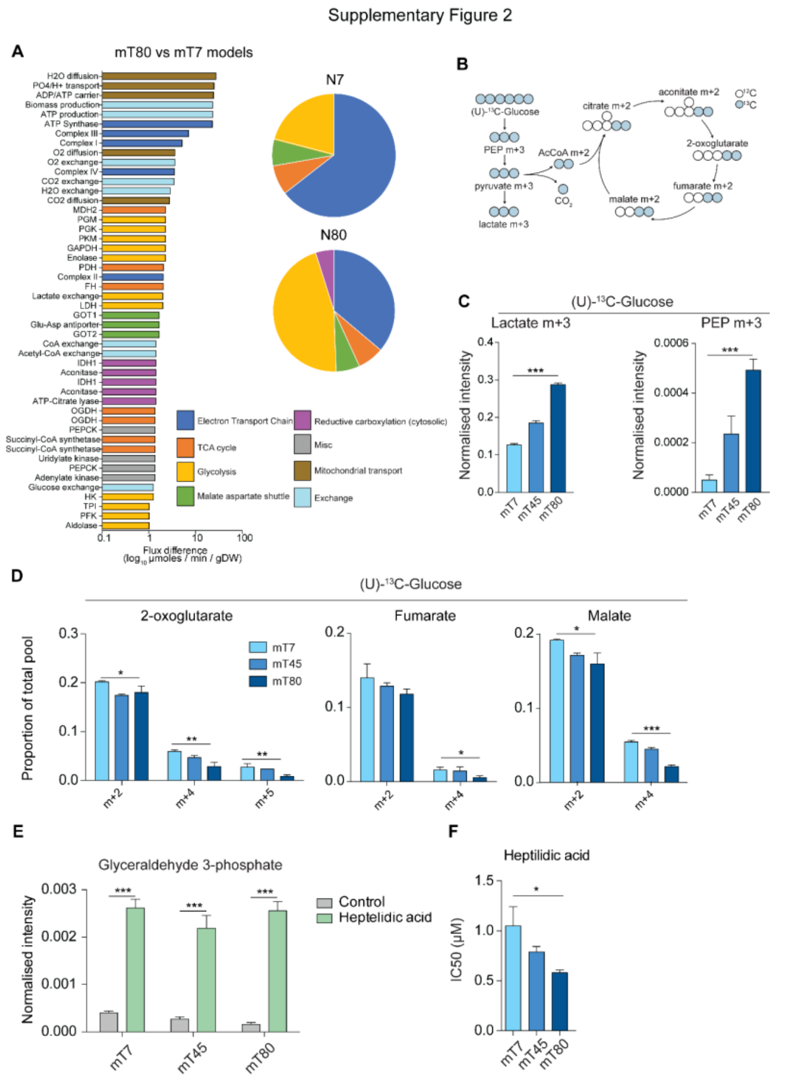

Supplementary Figure 2 related to Figure 2. Metabolic features of mTUNE cells. (A) Top 10% affected reactions between mT80 and mT7 as predicted by metabolic modelling. Flux difference between mT80 and mT7 model is shown in log_{10} scale. Each reaction is colour-coded based on distribution into metabolic pathways. Duplicated reactions indicate intermediate of reactions. (B) Schematic representation of metabolite labelling pattern from (U)-^{13}C-glucose. (C) Normalised intensities (total metabolite count) of lactate m+3 (left) and phosphoenolpyruvate (PEP) m+3 (right) after incubation with (U)-^{13}C-glucose. (D) Labelling patterns of the indicated TCA cycle intermediates after incubation with (U)-^{13}C-glucose. (E) Normalised intensity (total metabolite count) of glyceraldehyde 3-phosphate (GA3P) upon treatment of mT7, mT45 and mT80 cells with vehicle control or 0.5 μM of the GAPDH inhibitor heptelidic acid. Data are mean ± s.e.m. from 3 independent cultures. (F) IC50 values of heptelidic acid on the proliferation of mT7, mT45 and mT80 cells. All data are mean ± s.e.m. from at least three independent cultures. (C-D, F) *, ** and *** indicate one-way ANOVA p-value ≤ 0.05, 0.001 and 0.001, respectively. (E) *** indicates two-sided t-test p-value ≤ 0.001.

Supplementary Figure 3

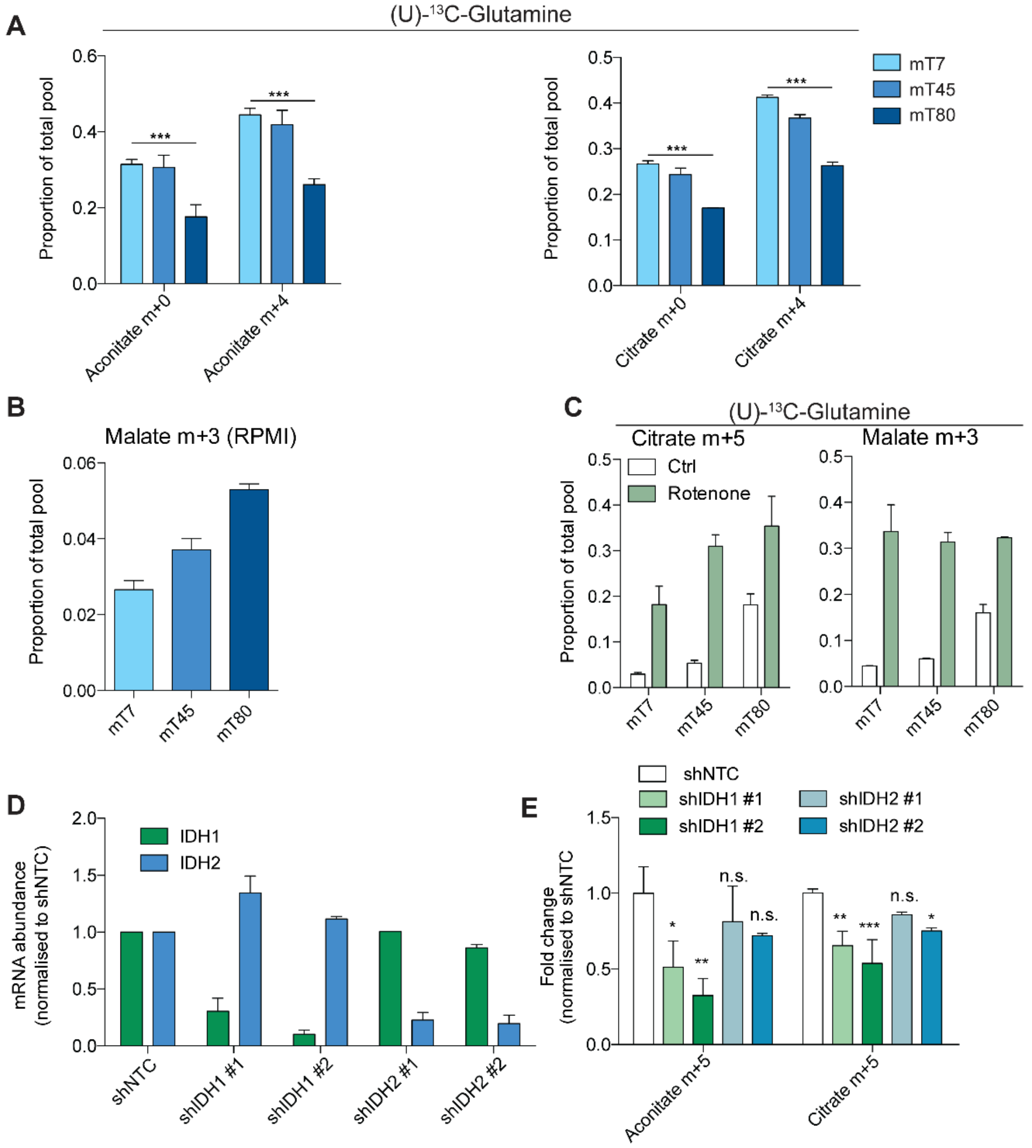

Supplementary Figure 3 related to Figure 2. Assessment of reductive carboxylation in the cybrid cells. (A) Labelling patterns of the indicated TCA cycle intermediates after incubation with U-^{13}C-glutamine. (B) Proportion of total pool of malate m+3 originating from U-^{13}C-glutamine in mT7, mT45 and mT80 cells grown in RPMI medium. Data are mean ± s.d. from one representative experiment. (C) Proportion of total pool of citrate m+5 and malate m+3 originating from U-^{13}C-glutamine in mT7, mT45 and mT80 cells in the presence of 0.5 µM rotenone. Data are mean ± s.d. from one representative experiment. (D) mRNA expression of IDH1 and IDH2 genes upon selective knock down of IDH1 or IDH2 with two independent shRNA constructs. Actin mRNA expression was used as endogenous control and data were normalised on shNTC control. Data are mean ± s.d. from one representative experiment. (E) Levels of metabolites from reductive carboxylation of U-^{13}C-Glutamine in mT80 cells infected with shRNA constructs targeting IDH1 or IDH2. Data are normalised on non-targeting shRNA control (shNTC). (A, E) Data are mean ± s.e.m. from three independent cultures. (A) *** indicates one-way ANOVA p-value ≤ 0.001. (C) *, **, *** indicate Dunnett's p-value ≤ 0.05, 0.01 and 0.001, respectively. n.s. = not significant.

Supplementary Figure 4

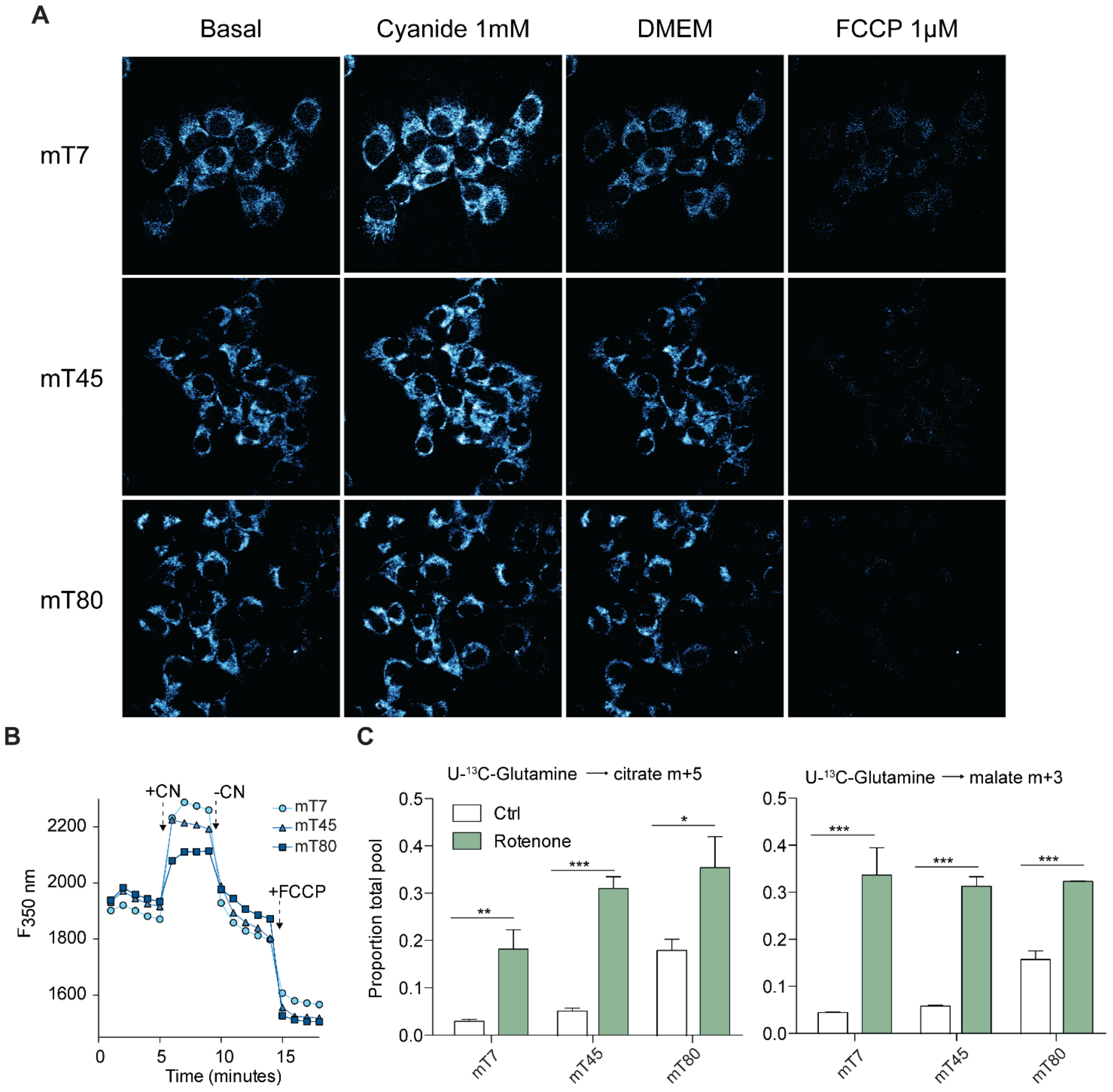

Supplementary Figure 4 related to Figure 3. Mitochondrial NADH in the cybrid cells. (A) Representative images of mT7, mT45 and mT80 cells excited at 350 nm. (B) Fluorescence intensity after excitation at 350 nm extracted from image time series analysis of mT7, mT45 and mT80 cells in basal conditions or after addition of 1 mM cyanide (CN), medium replenishment and addition of 1 μM FCCP. (C) Proportion of total pool for citrate m+5 and malate m+3 in mT7, mT45, and mT80 cells treated with vehicle control or 0.5 μM rotenone. Data are mean ± s.e.m. from three independent cultures. *, ** and *** indicate one-way ANOVA p-value ≤ 0.05, 0.001 and 0.001, respectively.

Supplementary Figure 5

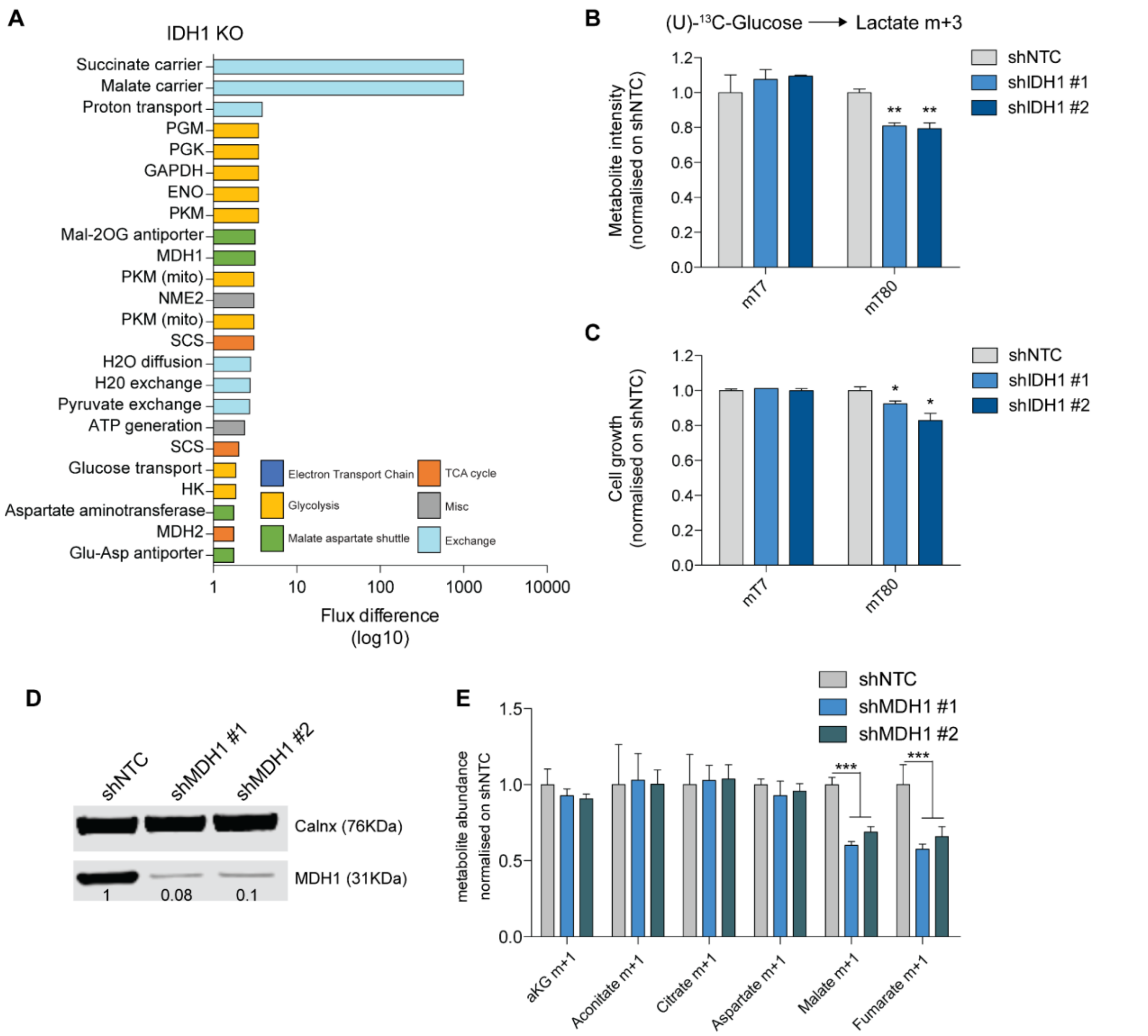

Supplementary Figure 5 related to Figure 4. The role of reductive carboxylation in the cybrid cells. (A) Top 10% affected reactions between mT80 and mT7 as predicted by metabolic modelling. Each reaction is colour-coded based on distribution into metabolic pathways. (B) Levels of lactate secretion in mT7 and mT80 cells upon silencing of IDH1. Data are normalised on shNTC controls and are ± s.e.m. from three independent cultures. (C) Cell growth of mT7 and mT80 cells upon silencing of IDH1. Data are normalised on shNTC controls and are ± s.e.m. from three independent cultures. (D) Western blot analysis of MDH1 expression upon infection of mT80 cells with non-targeting control (shNTC) or shRNA constructs targeting MDH1 (shMDH1 #1 and #2). Densitometry values are shown. Representative blot of three independent experiments. (E) Levels of metabolites from 1-^{13}C-Glutamine in shMDH1 mT80 cells. Data are normalised on non-targeting shRNA control (shNTC). *, **, ** indicates two-sided t-test p-value ≤ 0.05, 0.01 and 0.001,

Supplementary Figure 6

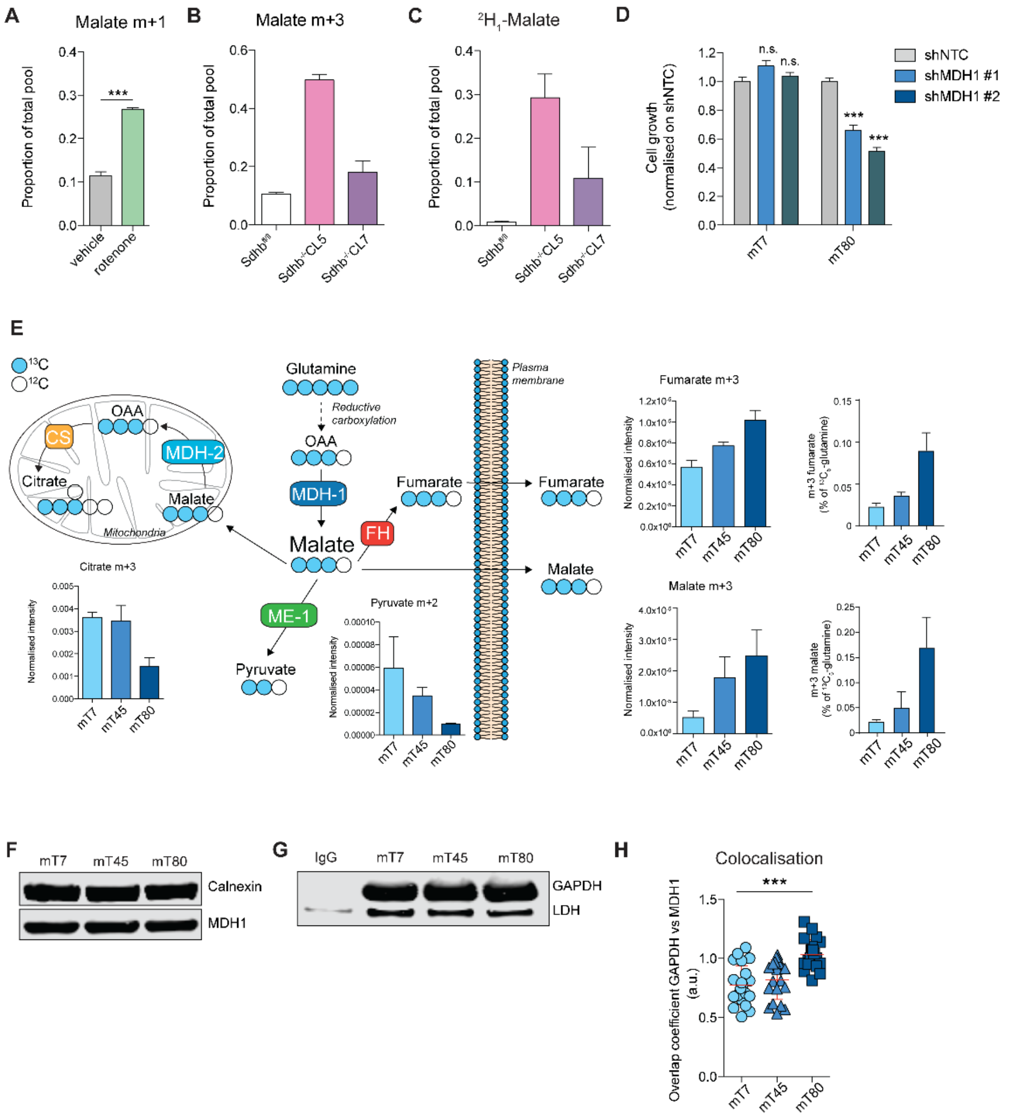

Supplementary Figure 6 related to Figure 4. Functional interaction between MDH1 and GAPDH. (A) Proportion of total pool for malate m+1 in mT7 cells treated with vehicle control or 0.5 μM rotenone and incubated with 1-^{13}C-glutamine. (B-C) Proportion of total pool of malate m+3 (B) and ^{2}H-malate in SDH null cells grown in the presence of (U)-^{13}C-glutamine (B) or 4-^{2}H-glucose (C). Data are mean ± s.d. from one representative experiment. (D) Cell growth of mT7 and mT80 cells upon silencing of MDH1. Data are normalised on shNTC controls and are ± s.e.m. from three independent cultures. (E) Intracellular levels of citrate m+3 and pyruvate m+2, and extracellular levels of malate m+3 and fumarate m+3 in mTUNE cells grown in the presence of (U)-^{13}C-glutamine. The percentage of m+3 fumarate and malate produced vs glutamine consumed (moles released/consumed in 24 hours), are indicated. Data are mean ± s.d. from one representative experiment. (F) Western blot analysis of MDH1 expression in mT7, mT45, and mT80 cells. Calnexin was used as loading control. (G) Immunoprecipitation analysis of GAPDH in mT7, mT45 and mT80 cells. Mouse IgG were used as isotype control for the IP reaction. (H) Co-localisation of fluorescence signals from GAPDH and MDH1 as detected in mT7, mT45, and mT80 by immunofluorescence. Data are mean ± s.d. from 20-30 ROIs per conditions. (A, D) *** indicates two-sided t-test p-value ≤ 0.001. (H) *** indicates one-way ANOVA p-value ≤ 0.001.

Supplementary Figure 7

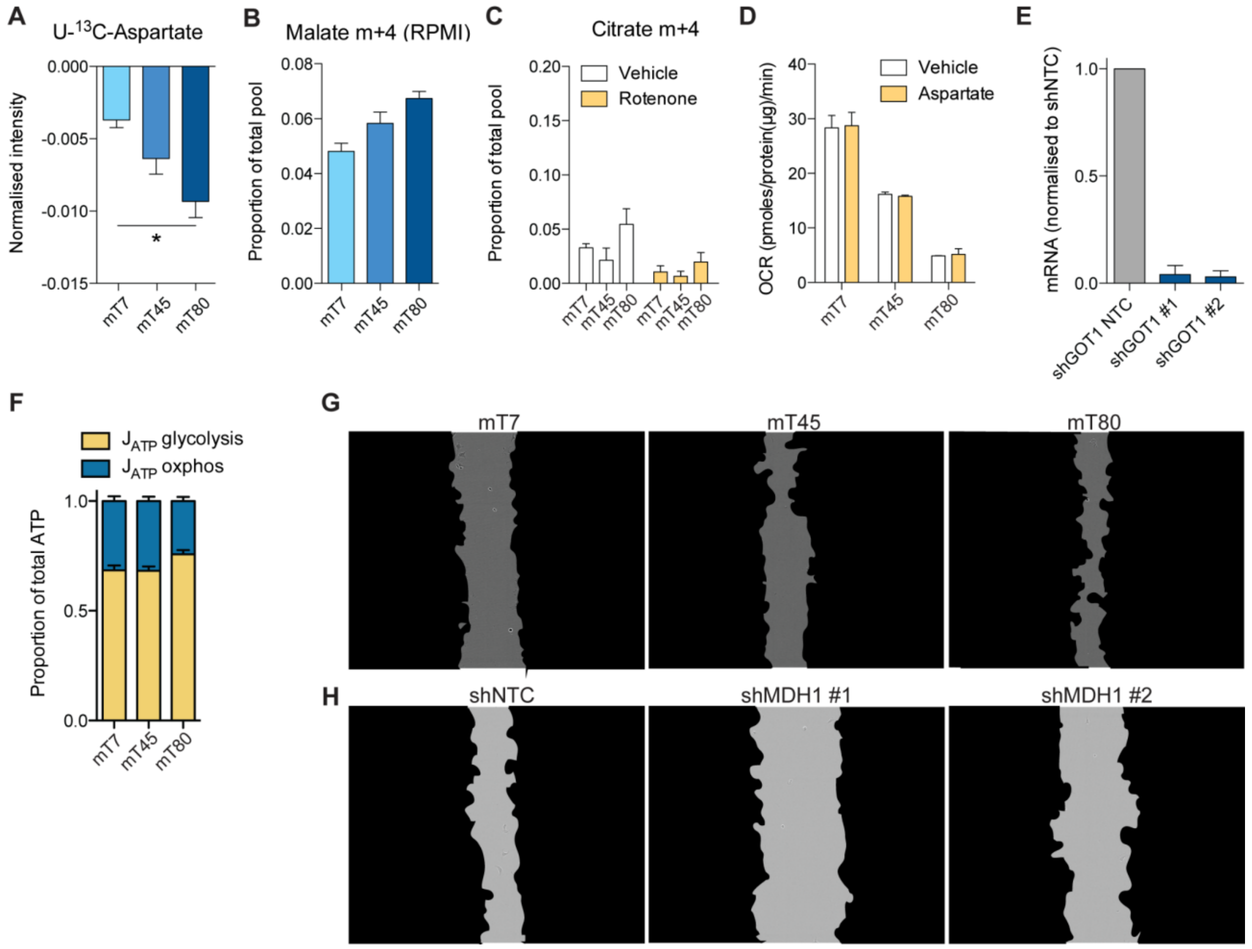

Supplementary Figure 7 related to Figure 5. Aspartate metabolism in the cybrid cells. (A) Consumption of (U)-^{13}C-aspartate from extracellular medium in mTUNE cells. * indicates one-way ANOVA p-value ≤ 0.05. (B) Proportion of total pool of malate m+4 upon supplementation with (U)-^{13}C-aspartate in mTUNE cells grown in RPMI medium. (C) Proportion of total pool for citrate m+4 in mT7, mT45 and mT80 cells treated with vehicle control or 0.5 µM rotenone and incubated with U-13C-aspartate. (D) Oxygen consumption rate (OCR) of mT7, mT45, and mT80 cells upon addition of vehicle control or 5 mM aspartate. (E) mRNA levels of GOT1 in mT80 shGOT1 cells. Data are mean ± s.d. from one representative experiment. (F) Proportion of total ATP originating from glycolysis and oxidative phosphorylation in mT7, mT45, and mT80 cells, as calculated from OCR and ECAR measurements. (G-H) Representative images of wound-healing assay 6 hours after application of wound in mT7, mT45, and mT80 (G) and shMDH1 (H) cells.
