# Supplementary material for: NADH Shuttling Couples Cytosolic Reductive Carboxylation of Glutamine with Glycolysis in Cells with Mitochondrial Dysfunction
Source: Mol Cell. 2018 Feb 15;69(4):581–593.e7. doi: 10.1016/j.molcel.2018.01.034 (PMC5823973; doi:10.1016/j.molcel.2018.01.034)
Supplement: Document S2. Article plus Supplemental Information [file mmc6.pdf]

Molecular Cell

Article

NADH Shuttling Couples Cytosolic Reductive Carboxylation of Glutamine with Glycolysis in Cells with Mitochondrial Dysfunction

Graphical Abstract

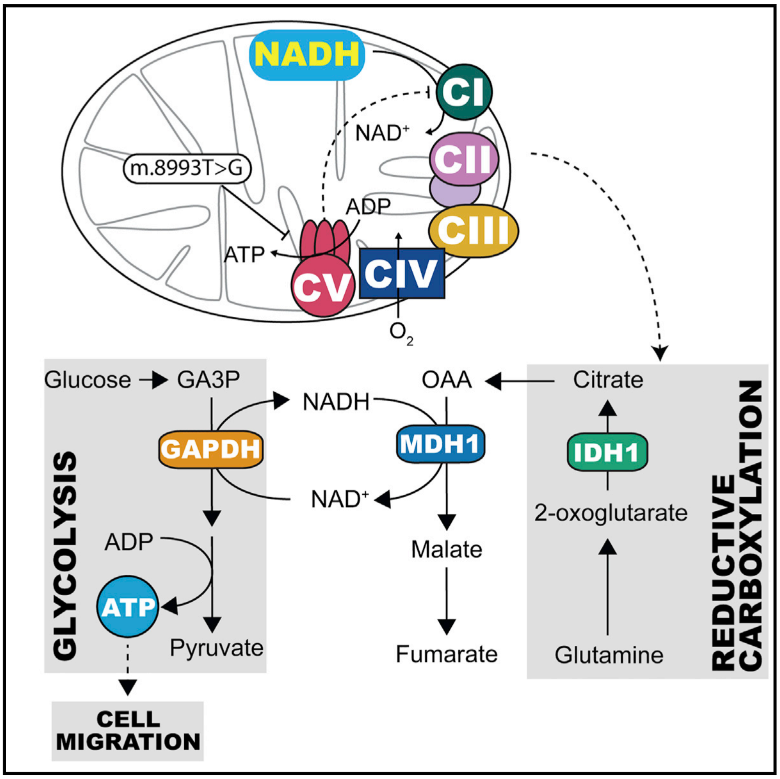

Authors

Edoardo Gaude, Christina Schmidt, Payam A. Gammage, ..., Gyorgy Szabadkai, Michal Minczuk, Christian Frezza

Correspondence

cf366@mrc-cu.cam.ac.uk

In Brief

The connection between mitochondrial dysfunction and changes in cell metabolism is poorly understood. Capitalizing on a model of mitochondrial DNA mutation, we found that impaired mitochondrial metabolism leads to cytosolic reductive carboxylation of glutamine, which regenerates NADH via malate dehydrogenase to fuel glycolysis. This pathway also supports cell migration.

Highlights

- mTUNE is a new model of tunable mitochondrial dysfunction
- Metabolic model predicts link between reductive carboxylation and glycolysis in mTUNE
- Reductive carboxylation is regulated by mitochondrial NADH redox state
- MDH1 couples reductive carboxylation to glycolysis and supports cell migration

Gaude et al., 2018, Molecular Cell *69*, 581–593
February 15, 2018 © 2018 The Author(s). Published by Elsevier Inc.
https://doi.org/10.1016/j.molcel.2018.01.034

CellPress

CellPress

NADH Shuttling Couples Cytosolic Reductive Carboxylation of Glutamine with Glycolysis in Cells with Mitochondrial Dysfunction

Edoardo Gaude,[1] Christina Schmidt,[1] Payam A. Gammage,[2] Aurelien Dugourd,[3] Thomas Blacker,[4] Sew Peak Chew,[5] Julio Saez-Rodriguez,[3,6] John S. O'Neill,[5] Gyorgy Szabadkai,[4,7,8] Michal Minczuk,[2] and Christian Frezza[1,9,*]
[1]Medical Research Council Cancer Unit, University of Cambridge, Hutchison/MRC Research Centre, Box 197, Cambridge Biomedical Campus, Cambridge CB2 0XZ, UK
[2]Medical Research Council Mitochondrial Biology Unit, University of Cambridge, Wellcome Trust/MRC Building, Hills Road, Cambridge CB2 0XY, UK
[3]Joint Research Centre for Computational Biomedicine, Faculty of Medicine, RWTH Aachen University, 52074 Aachen, Germany
[4]Research Department of Cell and Developmental Biology, University College London, London WC1E 6BT, UK
[5]MRC Laboratory of Molecular Biology, Francis Crick Avenue, Cambridge CB2 2QH, UK
[6]European Molecular Biology Laboratory, European Bioinformatics Institute (EMBL-EBI), Hinxton, UK
[7]Department of Biomedical Sciences, University of Padua and CNR Neuroscience Institute, Padua 35121, Italy
[8]The Francis Crick Institute, Midland Road, London NW1 1AT, UK
[9]Lead Contact
*Correspondence: cf366@mrc-cu.cam.ac.uk
https://doi.org/10.1016/j.molcel.2018.01.034

SUMMARY

The bioenergetics and molecular determinants of the metabolic response to mitochondrial dysfunction are incompletely understood, in part due to a lack of appropriate isogenic cellular models of primary mitochondrial defects. Here, we capitalize on a recently developed cell model with defined levels of m.8993T>G mutation heteroplasmy, mTUNE, to investigate the metabolic underpinnings of mitochondrial dysfunction. We found that impaired utilization of reduced nicotinamide adenine dinucleotide (NADH) by the mitochondrial respiratory chain leads to cytosolic reductive carboxylation of glutamine as a new mechanism for cytosol-confined NADH recycling supported by malate dehydrogenase 1 (MDH1). We also observed that increased glycolysis in cells with mitochondrial dysfunction is associated with increased cell migration in an MDH1-dependent fashion. Our results describe a novel link between glycolysis and mitochondrial dysfunction mediated by reductive carboxylation of glutamine.

INTRODUCTION

Central carbon metabolism is regulated by the fine balance between mitochondrial function and glycolysis. The full oxidation of glucose through the mitochondrial tricarboxylic acid (TCA) cycle is a defining feature of eukaryotes and is tightly regulated by genetic and post-transcriptional control of enzyme activity as well as allosteric mechanisms (Cairns et al., 2011; Ros and Schulze, 2013). It has been observed that impairment of mitochondrial function by mutations in mitochondrial enzymes (Sciacovelli et al., 2014) or by pharmacological inhibition of the respiratory chain (RC) (Dickman and Mandel, 1990) leads to activation of glycolysis. This switch toward glycolysis has been observed in most cancer cells even in the presence of oxygen, a phenomenon known as aerobic glycolysis, and it is thought to contribute to the increased demand for biosynthetic intermediates generated from glucose (Pavlova and Thompson, 2016). Although the link between mitochondrial dysfunction and aerobic glycolysis has been extensively reported, its molecular underpinnings remain poorly characterized.

Mitochondrial dysfunction has been associated with induction of reductive carboxylation, an alternative pathway for glutamine catabolism that supports biosynthesis of lipids (Metallo et al., 2011) and nucleotides (Birsoy et al., 2015; Sullivan et al., 2015), as well as mitochondrial redox state (Jiang et al., 2016). Yet the metabolic determinants of the activation of reductive carboxylation remain elusive. Moreover, it is unclear whether reductive carboxylation that results from impaired mitochondrial function contributes to the rewiring of glycolytic metabolism.

An obstacle to clarifying the link between mitochondrial function and central carbon metabolism is the difficulty of disentangling the direct consequences of dysregulated mitochondrial metabolism from secondary and indirect effects caused by mitochondrial defects. Cytoplasmic hybrids (cybrids) are established models to investigate the effects of primary mitochondrial dysfunction on cell physiology. Cytoplasts with wild-type or mutated mitochondrial DNA (mtDNA) are fused with the nucleus from a donor cell to evaluate the effect of a specific mtDNA mutation. However, cybrid generation is prone to artifacts. For instance, ethidium bromide used to eliminate mtDNA of the host cells also induces mutations in the nuclear genome. Moreover, the selection of individual clones often leads to unrepresentative clone-specific phenotypes, with marked interclonal heterogeneity being attributable to simple founder effects (King and Attardi, 1989; Martínez-Reyes

This is an open access article under the CC BY license (http://creativecommons.org/licenses/by/4.0/).

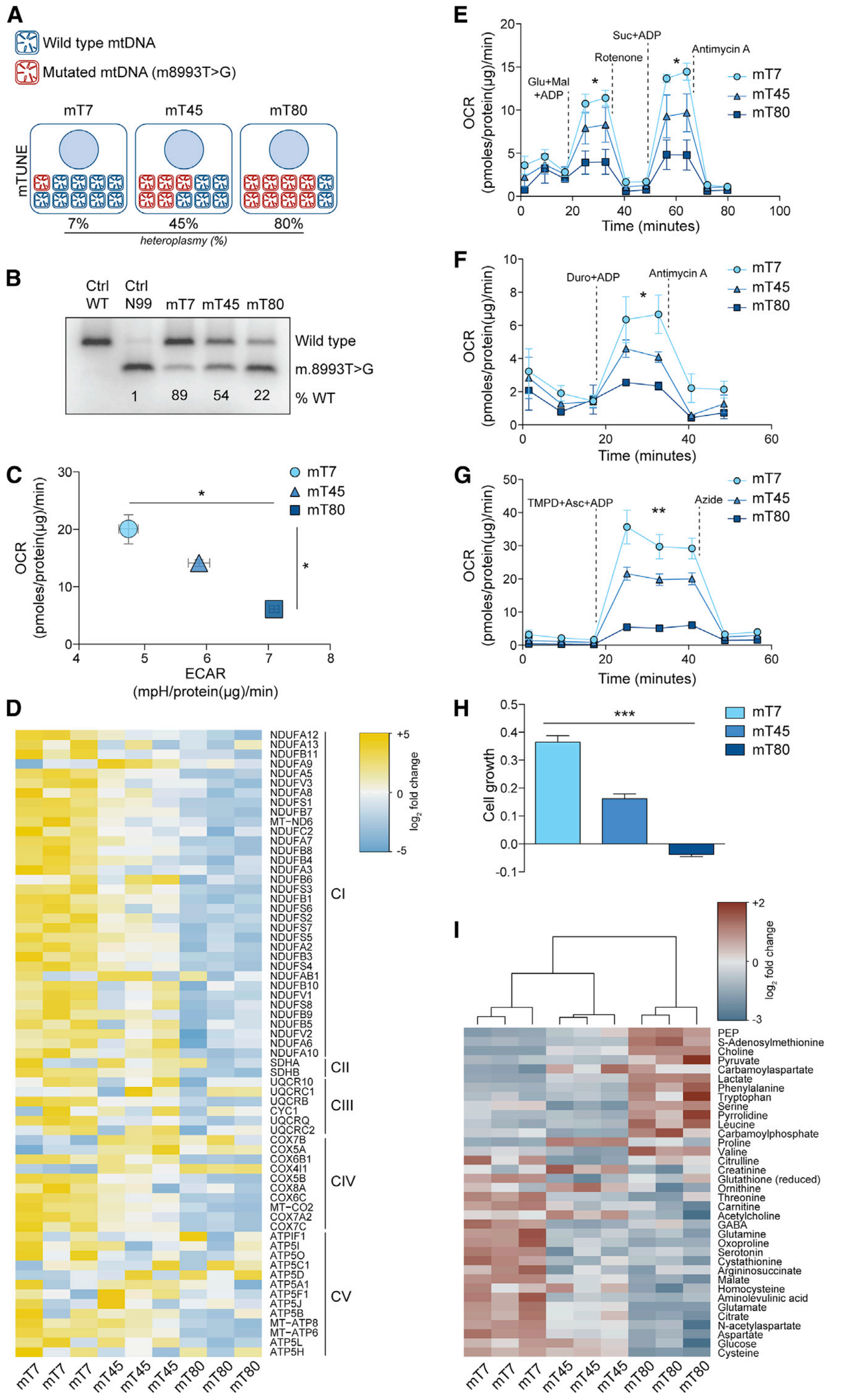

Figure 1. Increasing Levels of m8993T>G Mutation Are Associated with Changes in Mitochondrial Function

(A) Schematic representation of m8993T>G heteroplasmy in the mTUNE models.

(B) RFLP analysis of last-cycle hot PCR products (mtDNA nt positions 8339–9334) amplified from total DNA samples of 143B cells harboring indicated levels of m.8993T>G, obtained by treatment with mtZFNs. Wild-type cells and 99% m.8993T>G cybrids were used as controls.

(C) Basal extracellular acidification rate (ECAR) and oxygen consumption rate (OCR) in mT7, mT45, and mT80 cells. Data are normalized to protein content.

(D) Heatmap representation of mitochondrial respiratory complex protein expression in mT7, mT45, and mT80 cells as determined by mass spectrometry. Data represent values from three independent experiments and $\log_2$ fold change values are color-coded as indicated.

(E–G) Respiration of digitonin permeabilized mT7, mT45, and mT80 cells in the presence of (E) glutamate-malate (complex I) and succinate (complex II), (F) duroquinol (complex III), and (G) TMPD-ascorbate (complex IV).

(H) Proliferation of mT7, mT45, and mT80 cells in the presence of galactose instead of glucose. Cell growth was determined by calculating the slope of respective proliferation curves.

(I) Heatmap representation of significantly different intracellular metabolites (corrected ANOVA, $p < 0.05$) in mT7, mT45, and mT80 cells. Data represent values from three independent experiments and $\log_2$ fold change values are color-coded as indicated.

(C and E–H) Data are mean ± SEM from three independent cultures.

*$p \leq 0.05$, **$p \leq 0.01$, ***$p \leq 0.001$, one-way ANOVA.

et al., 2016). To overcome these issues, the selective elimination of mutated mtDNA with mitochondrially targeted zinc-finger nucleases (mtZFNs) has been recently used to generate isogenic cell lines with different levels of heteroplasmy of the mtDNA mutation m8993T>G. This mutation affects ATP6, a key subunit of ATP synthase, leading to neuropathy, ataxia, retinitis pigmentosa (NARP) syndrome, and fatal childhood maternally inherited Leigh's syndrome (MILS) (Gammage et al., 2014, 2016a). We therefore used this model of tunable mitochondrial dysfunction to investigate how mitochondrial function affects central carbon metabolism.

RESULTS

"mTUNE": A Model of Tunable Mitochondrial Dysfunction

To investigate the effects of primary mitochondrial dysfunction on cellular metabolism, we used a panel of isogenic cell lines in which heteroplasmy of the mtDNA mutation m8993T>G was shifted by treatment with mtZFNs (Gammage et al., 2016a). This model allows the generation of isogenic cell lines, hereafter called mTUNE (mT), with defined and stable low (7%, mT7), medium (45%, mT45), or high (80%, mT80) levels of m8993T>G heteroplasmy (Figures 1A and 1B). While growth

rate under basal conditions was comparable among cell types (Figure S1A), basal oxygen consumption rate (OCR) dramatically decreased proportionally to the levels of m8993T>G heteroplasmy (Figure 1C). Of note, the reduction in OCR was not caused by differences in mitochondrial mass among cell types (Figures S1B–S1D). Parallel to the decrease in OCR, we observed an increase in the extracellular acidification rate (ECAR) proportional to the levels of m8993T>G heteroplasmy (Figure 1C), consistent with the expected switch toward glycolysis in cells with mitochondrial dysfunction.

We then investigated in more detail how the m8993T>G mutation affects RC activity. A proteomic analysis revealed that, together with low levels of ATP6 and other subunits of ATP synthase, the abundance of most RC components is decreased in mT80 compared to mT45 and mT7 cells (Figure 1D). In line with this finding, the activity of individual RC complexes decreased in correlation with the level of heteroplasmy (Figures 1E–1G). Consistent with the presence of mitochondrial dysfunction, mT80 cells failed to grow in galactose (Figure 1H), a substrate whose slower catabolism requires compensatory activation of mitochondria for ATP generation (Marroquin et al., 2007).

We then performed steady-state metabolomics by liquid chromatography-mass spectrometry (LC-MS) on our cell lines to further investigate the metabolic changes caused by m8993T>G mutation. Hierarchical clustering of intracellular metabolite levels grouped together cell lines according to mtDNA heteroplasmy (Figure 1I). Importantly, we observed that levels of the glycolytic metabolites phosphoenolpyruvate (PEP), pyruvate, and lactate were increased in mT80, while metabolites linked with mitochondrial function, such as aspartate (Birsoy et al., 2015; Sullivan et al., 2015), citrate, and malate, were decreased, compared to mT7 and mT45 (Figure 1I). To gain a deeper understanding of the metabolic response to mitochondrial dysfunction, we measured the consumption and release of extracellular metabolites in our mTUNE models (Table S1). In line with ECAR measurements, we found that lactate secretion was increased in mT80, compared to mT7 and mT45 (Figure S1E). Together, these data indicate that the mTUNE models exhibit a degree of mitochondrial dysfunction proportional to the level of heteroplasmy, which results in distinct and stable metabolic configurations. Therefore, this model represents an ideal setting to investigate the consequences of a primary mitochondrial dysfunction on cellular metabolism.

Constraint-Directed Metabolic Modeling Predicts Association between Cytosolic Reductive Carboxylation and Glycolysis

To systematically investigate the metabolic changes associated with mitochondrial dysfunction in these cells, we took advantage of a recently published *in silico* metabolic model that provides a detailed reconstruction of mitochondrial and central carbon metabolism reactions (Zieliński et al., 2016). We refined this model by including consumption and release rates of metabolites (Table S1) and by constraining RC activity with RC complex-dependent measurements of OCR (Figures 1E–1G; Table S2). We then compared the predicted metabolic fluxes in mT7 and mT80. Besides the expected changes in RC activity, oxygen exchange, and ATP production, the model predicted an increase in several glycolytic reactions and decreased activity of multiple enzymes of the TCA cycle and malate-aspartate shuttle (MAS) in mT80 cells (Figures 2A and S2A). Interestingly, the model predicted activation of cytosolic reductive carboxylation of glutamine in mT80 cells, while this pathway is inactive in mT7 cells (Figure 2A). To assess the validity and robustness of our predictions, we investigated alternative solutions to reaction fluxes by performing flux variability analysis (FVA) (Mahadevan and Schilling, 2003). This analysis confirmed the uniqueness of reaction flux solutions predicted for, among others, glycolysis, MAS, and cytosolic reductive carboxylation (Table S3).

To experimentally test the predictions of the model, we cultured cells in the presence of uniformly labeled (U)-^{13}C-glucose (Figure S2B) and (U)-^{13}C-glutamine (Figure 2B) and assessed by LC-MS the labeling profile of downstream metabolites. We observed increased levels of ^{13}C-PEP and ^{13}C-lactate, and decreased levels of ^{13}C-labeled TCA cycle intermediates, such as 2-oxoglutarate, fumarate, and malate, in mT80 cells (Figures S2C and S2D) upon incubation with (U)-^{13}C-glucose. Consistent with an increased dependency on glycolysis, mT80 cells were more sensitive to inhibition of GAPDH by heptelidic acid (Figure S2E), compared with mT7 (Figure S2F). The incubation of cells with (U)-^{13}C-glutamine (see Figure 2B for a schematic) revealed changes in glutamine oxidation in mT80, compared to mT45 and mT7 cells. In particular, we observed a decrease in m+4 isotopologues of citrate and aconitate, consistent with reduced oxidation of glutamine via the TCA cycle (Figure S3A). We also observed a substantial increase in aconitate and citrate m+5, and in malate and fumarate m+3 in mT80 cells compared to mT7 and mT45 (Figure 2C), indicative of reductive carboxylation of glutamine proportional to level of heteroplasmy. Of note, this metabolic rewiring was observed even when cells were cultured in medium with a different composition (Figure S3B), indicating that these metabolic changes are robust under different conditions. To further confirm the link between mitochondrial dysfunction and reductive carboxylation, we performed (U)-^{13}C-glutamine tracing in the presence of the complex I-specific inhibitor rotenone. Consistently, rotenone led to increased contribution of reductive glutamine metabolism to citrate and malate pools in all our cell lines (Figure S3C).

To assess whether induction of reductive carboxylation in mT80 cells occurred in the cytosolic or mitochondrial compartment, we silenced either the cytosolic isocitrate dehydrogenase (IDH), *IDH1*, or the mitochondrial isoform, *IDH2*, in mT80 cells (Figure S3D). We then followed incorporation of (U)-^{13}C-glutamine carbons into downstream metabolites using LC-MS. Accumulation of aconitate and citrate m+5 was markedly reduced when *IDH1* was suppressed, while downregulation of *IDH2* had only minor effects (Figure S3E). These data are in line with the predictions of the metabolic model and suggest that mitochondrial dysfunction induces a glycolytic switch, triggering cytosolic reductive carboxylation.

Reductive Carboxylation Is Regulated by NAD$^+$/NADH Ratio

We then investigated the possible determinants of cytosolic reductive carboxylation triggered by mitochondrial dysfunction. Reductive carboxylation has been associated with altered levels of NAD$^+$/NADH ratio (Fendt et al., 2013), although it is not clear whether these changes are sufficient to drive reductive

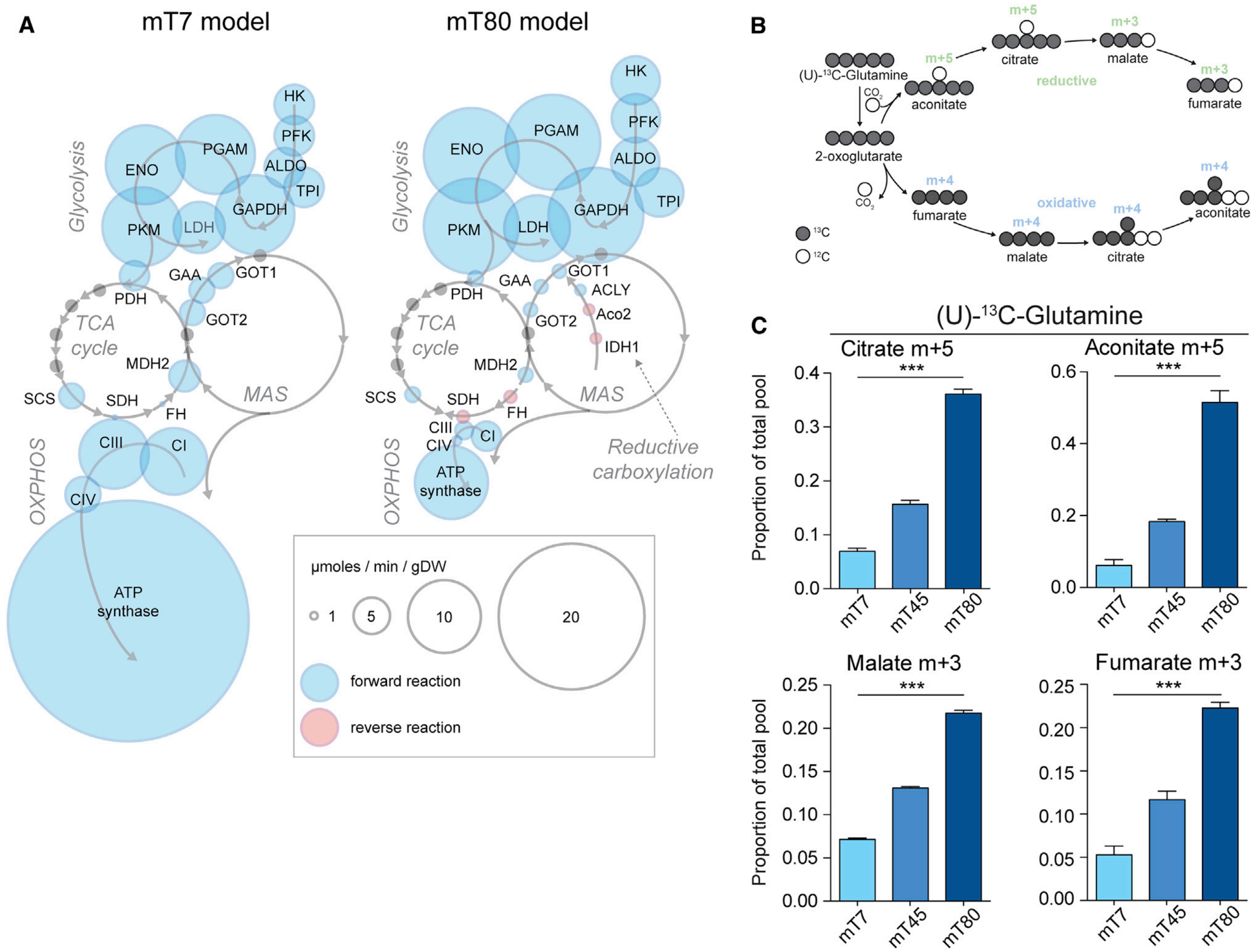

Figure 2. Mitochondrial Function of mT7, mT45, and mT80 Cells Is Associated with Induction of Reductive Carboxylation in the Cytosol

(A) Bubble representation of reactions involved in glycolysis, respiration, MAS, and cytosolic reductive carboxylation as predicted by mT7 and mT80 metabolic models. Bubble size is indicative of predicted reaction flux (μmoles/min/gDW). Blue and red bubbles indicate forward and reverse reactions. Gray arrows show the predicted direction of reactions, while gray dots represent reactions present in the depicted pathways, but with no predicted flux change.

(B) Schematic representation of metabolite labeling pattern from (U)-^{13}C-glutamine. Gray circles indicate ^{13}carbon.

(C) Proportion of total pool of metabolites originating from reductive carboxylation of U-^{13}C-glutamine; aconitate m+5, citrate m+5, malate m+3, and fumarate m+3 are shown. Data are mean ± SEM from three independent cultures. ***$p \leq 0.001$, one-way ANOVA.

carboxylation. To investigate whether mitochondrial function affects NAD$^+$/NADH ratio in our cell lines, we measured total cellular NAD$^+$/NADH levels using an enzymatic assay, and mitochondria-specific NAD(P)H using confocal microscopy (Blacker and Duchen, 2016). NAD$^+$/NADH ratio was significantly lower in mT80 cells, compared with mT45 and mT7 (Figure 3A), and it correlated with decreased NAD(P)H oxidation in mitochondria (Figures 3B, S4A, and S4B). These results indicate that impairment of respiratory activity in mT80 cells alters NADH oxidation in mitochondria, leading to a decreased total cellular NAD$^+$/NADH ratio. Of note, reduced availability of NAD$^+$, by altering malate dehydrogenase 2 (MDH2) activity, could explain the suppression of MAS predicted by our metabolic model.

To further assess the role of NAD$^+$/NADH balance in our cell lines, we rescued mitochondrial NADH oxidoreductase activity by expressing yeast-derived NADH dehydrogenase internal (NDI)-1 in mT80 cells (Figures 3C and 3D), where RC complex I activity is compromised. Expression of NDI-1 restored basal respiration, which was partially resistant to rotenone, but not to antimycin A (Figure 3E), consistent with the lack of sensitivity of NDI-1 to rotenone. Importantly, NAD$^+$/NADH ratio increased upon expression of NDI-1 (Figure 3F). To assess whether NDI-1 affected glutamine reductive metabolism, we performed 1-^{13}C-glutamine labeling, which selectively tracks reductive carboxylation (Metallo et al., 2011). Consistent with our hypothesis, NDI-1 expression diminished reductive metabolism (Figure 3G). In further support of a causative link between mitochondrial dysfunction, changes in NAD$^+$/NADH ratio, and cytosolic reductive carboxylation, the RC complex I-specific inhibitor rotenone led to decreased glutamine oxidation and increased

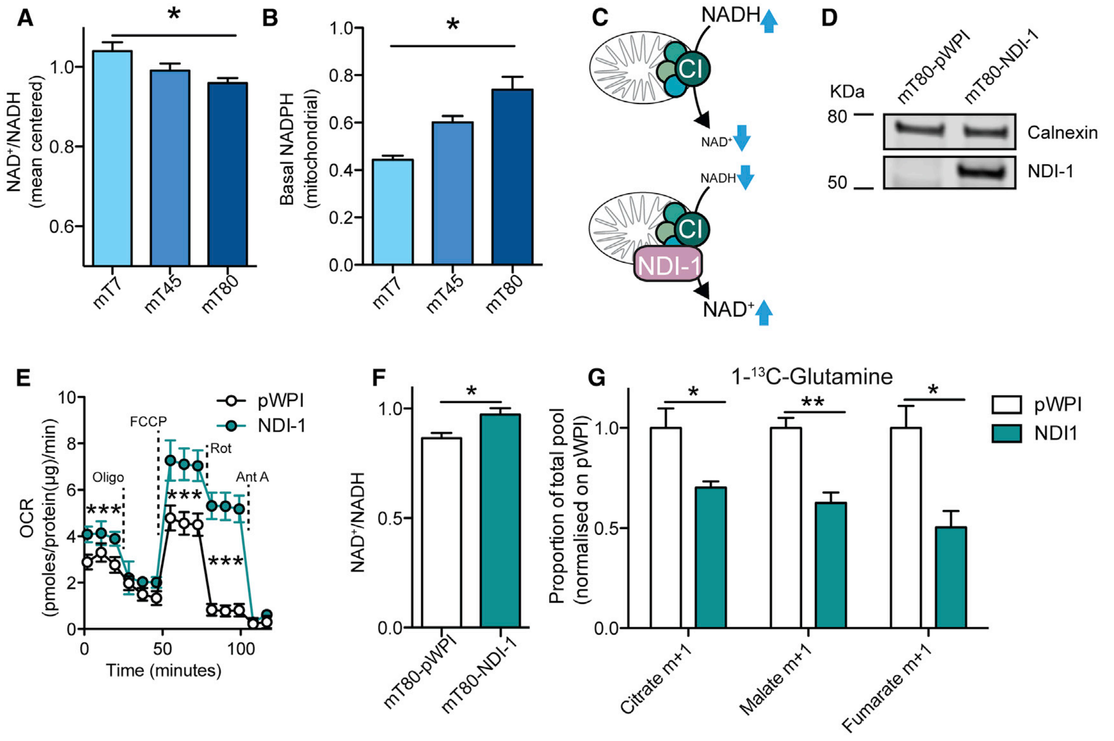

Figure 3. Reductive Carboxylation Is Dependent on NAD$^+$/NADH Levels

(A) Total levels of NAD$^+$/NADH in mT7, mT45, and mT80 cells measured using an enzymatic assay. Data are mean ± SEM from four independent experiments.

(B) Levels of basal mitochondrial NAD(P)H measured by NAD(P)H autofluorescence.

(C) Schematic representation of the rescue of mitochondrial NADH oxidoreductase activity by the expression of yeast-derived NADH dehydrogenase internal (NDI-1).

(D) Western blot analysis of NDI-1 expression upon infection of mT80 cells with pWPI control or NDI-1 lentiviral vectors. Representative blot of two independent experiments.

(E) OCR of pWPI and NDI-1 mT80 cells in basal conditions or after injection of oligomycin, FCCP, rotenone, and antimycin A. Data are mean ± SD from one representative experiment, performed with five replicates (out of two).

(F) Total levels of NAD$^+$/NADH in pWPI and NDI-1 mT80 cells. Data are mean ± SEM from three independent cultures.

(G) Proportion of total pool of citrate, malate, and fumarate in pWPI and NDI-1 mT80 cells grown in the presence of 1-^{13}C-glutamine. Data are mean ± SEM from three independent cultures.

*$p \leq 0.05$, one-way ANOVA (A and B). *$p \leq 0.05$, **$p \leq 0.01$, ***$p \leq 0.001$, two-sided t test (E–G).

reductive carboxylation (Figure S4C). Together, these data indicate that impairment of cellular NAD$^+$/NADH ratio by mitochondrial NADH turnover induces reductive carboxylation of glutamine.

Reductive Carboxylation Is Coupled with Glycolysis via MDH1

We then assessed the functional relevance of cytosolic reductive carboxylation in the metabolic reprogramming of our cells. To this end, we first simulated the suppression of *IDH1 in silico*, followed by computation of the changes in metabolic fluxes. Of note, the *in silico* depletion of *IDH1* led to significant changes to reactions belonging to glycolysis and MAS (Figure S5A). Indeed, among the top reactions affected by the suppression of reductive carboxylation were major glycolytic enzymes, such as GAPDH and pyruvate kinase (PK), as well as glutamate oxaloacetate transaminase 1 (GOT1) and MDH1, two components of MAS (Figure S5A). Interestingly, we found that the *in silico* deletion of *IDH1* led to a reduction of ATP yield in mT80 (Figure 4A) but had no effects in mT7. In support of this prediction, the silencing of IDH1 in mT80 cells led to decreased lactate secretion and cell proliferation, but it had no effects in mT7 (Figures S5B and S5C). Moreover, among the components of MAS, we observed a striking increase in the contribution of MDH1 to ATP production in mT80 compared with mT7 model (Figure 4A). These results suggest that cytosolic reductive carboxylation and MDH1 may be linked to glycolysis and subsequent ATP generation. In addition, these results are in line with the recent observation that MDH1 can support glycolysis via recycling of cytosolic NADH in proliferating cells (Hanse et al., 2017).

We investigated whether the synthesis of cytosolic malate via MDH1 could support glycolysis in cells with mitochondrial dysfunction. To this aim, we first assessed the functional consequences of the silencing of *MDH1* in mT80 cells (Figure S5D). The production of malate m+1 and fumarate m+1 from 1-^{13}C-glutamine was markedly reduced in MDH1-depleted mT80 cells, compared to non-targeting control (Figure S5E). These results confirmed our prediction that IDH1-dependent reductive carboxylation can generate cytosolic malate via MDH1 activity in mT80 cells.

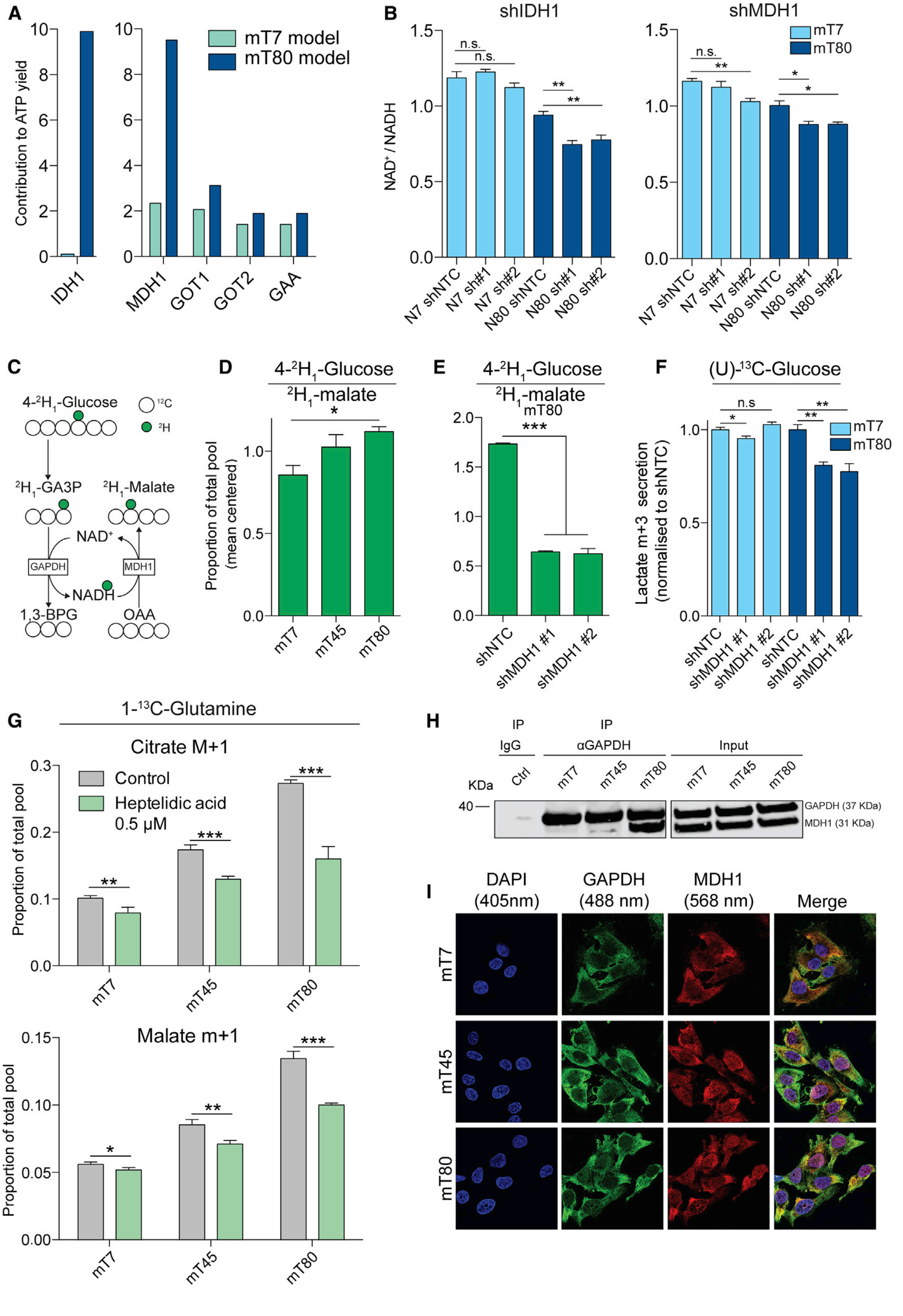

(legend on next page)

MDH1 generates NAD^+ upon reduction of oxaloacetate to malate. We therefore hypothesized that MDH1 could support glycolysis by providing NAD^+, a key cofactor required by GAPDH. Importantly, the silencing of either IDH1 or MDH1 affected NADH redox state in mT80 cells, but had little effect in mT7 cells (Figure 4B), suggesting that IDH1 and MDH1 play a role in NADH oxidation in cells with mitochondrial dysfunction. To further investigate the possible coupling between glycolytic NADH and MDH1, we performed a hydrogen tracing experiment with 4-^{2}H-glucose, which allows measurement of the transfer of hydrogen atoms from GAPDH-derived NADH to cytosolic metabolites (Figure 4C) (Lewis et al., 2014). We found that malate m+1 was increased in mT80 cells, compared with mT45 and mT7 (Figure 4D), confirming the functional coupling between GAPDH and MDH1, as well as the directionality of MDH1 (Figure 4E). Furthermore, we found that treatment with rotenone led to increased levels of malate m+1 in mT7 cells incubated with 4-^{2}H-glucose (Figure S6A), demonstrating that the coupling of MDH1 and GAPDH can be induced by pharmacological suppression of mitochondrial function. To expand our findings to additional cell models of mitochondrial dysfunction, we performed labeling experiments on a panel of *succinate dehydrogenase b* (*Sdhb*)-deficient mouse cell lines, which exhibit profound impairment of mitochondrial function (Cardaci et al., 2015). Of note, these cells exhibited reductive carboxylation of glutamine (Figure S6B) and showed increased levels of malate m+1 upon incubation with 4-^{2}H-glucose (Figure S6C), indicating that recycling of glycolytic NADH by MDH1 is a *bona fide* metabolic rewiring induced by mitochondrial dysfunction.

To assess whether MDH1 can support glycolytic flux, we performed (U)-^{13}C-glucose labeling and assessed lactate secretion in mT7 and mT80 cells upon silencing of *MDH1*. We observed decreased lactate secretion in mT80 cells when *MDH1* was silenced, while little or no effect was observed in mT7 cells (Figure 4F). Furthermore, we found that the silencing of MDH1 reduced cell proliferation of mT80 cells, but not of mT7 cells (Figure S6D). Together, these results suggest that MDH1 can support glycolysis and proliferation of cells with mitochondrial dysfunction.

To further corroborate the crosstalk between reductive carboxylation, MDH1 activity, and glycolysis, we assessed the contribution of reductive carboxylation to malate upon inhibition of GAPDH with heptelidic acid. Together with the expected accumulation of glyceraldehyde 3-phosphate (Figure S2E), we observed diminished levels of citrate and malate m+1 (Figure 4G) upon incubation with 1-^{13}C-glutamine, indicating that reductive carboxylation is intimately linked with GAPDH activity.

We then hypothesized that, to sustain uninterrupted flux through MDH1 and to avoid its accumulation, malate would be further converted to downstream metabolites. Malate could be metabolized via cytosolic malic enzyme 1 (ME1), producing pyruvate and NADPH; alternatively, malate could be shuttled into the mitochondria via MAS and enter the TCA cycle via oxidation through MDH2; finally, malate could be secreted into the medium (see Figure S6E for a schematic). In order to investigate the fate of malate generated by cytosolic reductive carboxylation, we performed U-^{13}C-glutamine tracing experiments and measured the levels of pyruvate, citrate, and extracellular malate. Interestingly, we observed reduced levels of pyruvate m+2 and citrate m+3 in mT80 cells, compared to mT7 and mT45 (Figure S6E), indicating that malate is not metabolized through ME1 and does not enter the CAC. Unexpectedly, we observed an increased secretion of malate m+3 in the medium of mT80 cells, indicating that malate generated via reductive carboxylation is secreted in the extracellular space (Figure S6E). Furthermore, we observed higher levels of extracellular fumarate m+3 in these cells, suggesting that malate can also be converted to fumarate by cytosolic fumarate hydratase (FH) and secreted out of the cell. Overall, these results suggest that increased cytosolic reductive carboxylation is coupled with secretion of malate and its product fumarate, likely to sustain MDH1 activity.

Finally, we hypothesized that the NADH shuttling between GAPDH and MDH1 could be facilitated by the physical interaction between the two enzymes, as observed for the interaction between LDH and GAPDH (Svedružić and Spivey, 2006). Using co-immunoprecipitation (coIP) assays, we found that GAPDH interacts with MDH1 (Figure 4H). Strikingly, we observed that mT80 cells displayed a greater interaction between GAPDH and MDH1, compared to mT45 and mT7 (Figure 4H). Importantly, increased interaction between GAPDH and MDH1 in mT80 cells was not due to higher protein expression of MDH1 in mT80 cells (Figure S6F). In addition, we observed interaction between GAPDH and LDH in mT7, mT45, and mT80 cells (Figure S6G), thus confirming previous evidence of formation of a complex between these two enzymes (Svedružić and Spivey, 2006). Finally, immunofluorescence experiments demonstrated increased co-localization between GAPDH and MDH1 in mT80 cells, compared to mT45 and mT7, thus further indicating an increased interaction between the two enzymes in the presence of mitochondrial dysfunction (Figures 4I and S6H).

Figure 4. Reductive Carboxylation Supports Glycolytic Flux via NADH Channeling between MDH1 and GAPDH

(A) Percentage of contribution to ATP production for the indicated enzymes, as predicted by metabolic modeling in mT80 and mT7 models.

(B) Total levels of NAD^+/NADH in shIDH1 and shMDH1 mT7 and mT80 cells, compared to shNTC controls, in basal conditions measured by enzymatic assay.

(C) Schematic representation of labeling pattern originating from 4-$^2H_1$-glucose. Deuterium atoms are represented as green filled circles.

(D and E) Proportion of total pool of malate m+1 originating from 4-$^2H_1$-glucose in mT7, mT45, and mT80 cells (D) and shMDH1 mT80 cells (E).

(F) Levels of secreted lactate m+3 upon incubation of shMDH1 mT7 and mT80 cells with (U)-^{13}C-glucose. Data are normalized to shNTC controls.

(G) Proportion of total pool for citrate m+1 and malate m+1 originating from 1-^{13}C-glutamine in mT7, mT45, and mT80 cells upon treatment with 0.5 μM of the GAPDH inhibitor heptelidic acid.

(H) GAPDH IP on lysates of mT7, mT45, and mT80 cells. The interaction between GAPDH and MDH1 is shown by coIP. Immunoglobulin G (IgG) is used in negative isotype controls. Representative images from two independent experiments.

(I) Immunofluorescence images of mT7, mT45, and mT80 cells stained with DAPI (blue), GAPDH (green), and MDH1 (red).

(B and D–G) Data are mean ± SEM from at least three independent cultures.

$^*p \leq 0.05$, $^{**}p \leq 0.01$, $^{***}p \leq 0.001$, two-sided t test (B and E–G). $^*p \leq 0.05$, one-way ANOVA (D). GAA, glutamate aspartate antiporter.

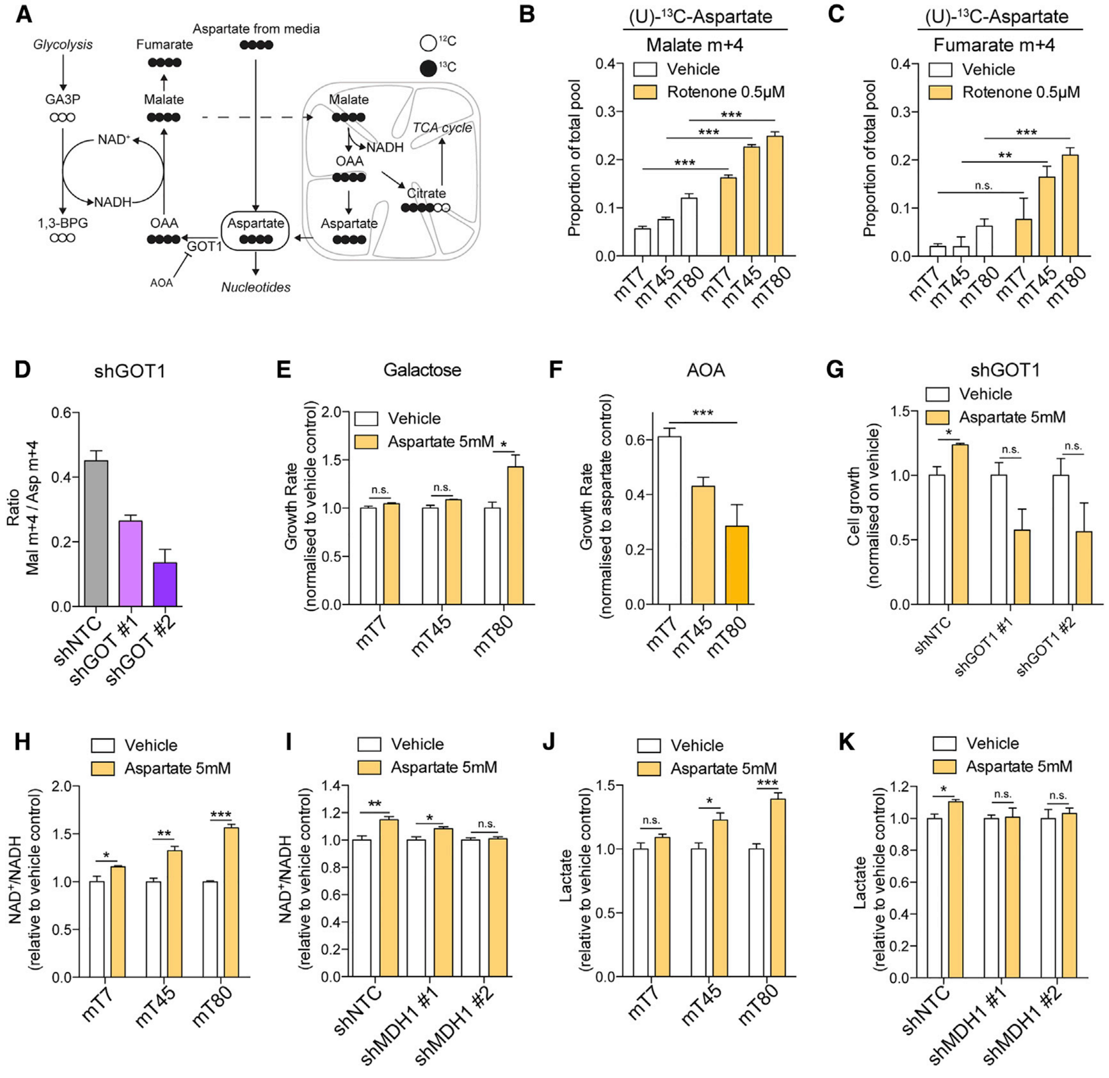

Figure 5. Aspartate Transamination Supports Flux through MDH1 and Generation of Malate

(A) Schematic representation of MAS and labeling patterns originating from (U)-^{13}C-aspartate.

(B and C) Proportion of total pool of malate m+4 (B) and fumarate m+4 (C) in mT7, mT45, and mT80 cells grown in the presence of U-^{13}C-aspartate upon treatment with vehicle control or 0.5 μM rotenone.

(D) Malate m+4 levels originating from (U)-^{13}C-aspartate in mT80 cells upon silencing of GOT1. Data are normalized to intracellular levels of aspartate m+4 and are mean ± SD from one independent experiment.

(E and F) Cell growth of mT7, mT45, and mT80 cells grown in 25 mM galactose and supplemented with 5 mM aspartate (E) upon treatment with 2 mM of the transaminase inhibitor aminooxyacetate (F). Data are normalized on cell growth of vehicle control (E) or on cell growth in the presence of aspartate only (F).

(G) Cell growth of mT80 cells grown in 25 mM galactose and supplemented with 5 mM aspartate upon silencing of GOT1. Data are normalized to the cell growth rate of vehicle control.

(H and I) Total levels of NAD^+/NADH in mT7, mT45, and mT80 cells (H) or shMDH1 mT80 cells (I) upon supplementation with 5 mM aspartate. Data are normalized on vehicle control.

(J and K) Secretion of lactate of mT7, mT45, and mT80 cells (J) or shMDH1 mT80 cells (K) upon supplementation with 5 mM aspartate. Data are normalized on vehicle control.

(B, C, and E–K) Data are mean ± SEM from at least three independent cultures.

*$p \leq 0.05$, **$p \leq 0.01$, ***$p \leq 0.001$, two-sided t test; n.s., not significant (B, C, E, and G–K). ***$p \leq 0.001$, one-way ANOVA (F).

Aspartate Supports Flux via MDH1 and Generates Malate

Our results indicate that the cytosolic component of the MAS, including GOT1, could function as an additional source of oxaloacetate for MDH1 (Figure 5A). To further investigate the functionality of the MAS in our models, cells were cultured in the presence of (U)-^{13}C-aspartate. Importantly, (U)-^{13}C-aspartate was taken up from the extracellular medium to a greater extent in mT80 cells, compared with mT7 and mT45 (Figure S7A). We found that aspartate is converted to m+4 malate and fumarate,

displaying the highest proportion of labeling in mT80, compared to mT45 and mT7 cells (Figures 5B and 5C). Furthermore, the accumulation of malate m+4 was also observed when cells were cultured in different media conditions (Figure S7B), suggesting that this reaction is independent from nutritional cues. Surprisingly, we could not detect labeled succinate, and the labeling of citrate from aspartate did not correlate with mitochondrial function and was not further increased by rotenone (Figure S7C), indicating that utilization of aspartate in our cell model is predominantly cytosolic. In support of this hypothesis, defects of cellular respiration in mT80 cells were not rescued by exogenous aspartate (Figure S7D). Furthermore, the treatment with rotenone led to increased m+4 labeling of malate and fumarate (Figures 5B and 5C) from (U)-^{13}C-aspartate, suggesting that contribution of aspartate to these metabolite pools is linked with mitochondrial function.

The synthesis of cytosolic malate from aspartate requires transamination to oxaloacetate (OAA), followed by reduction of OAA to malate via MDH1. Therefore, we reasoned that mT80 cells might be dependent on aspartate transamination for the regeneration of malate. To validate this hypothesis, we silenced the glutamate-oxaloacetate transaminase (GOT) 1 (Figure S7E), the enzyme responsible for aspartate transamination in the cytosol. The silencing of GOT1 led to decreased labeling of malate m+4 from ^{13}C-aspartate (Figure 5D), indicating that cytosolic aspartate transamination by GOT1 can support generation of malate. Finally, we showed that aspartate supplementation could rescue proliferation of mT80 cells grown in galactose, while only minor effects were observed in mT7 and mT45 cells (Figure 5E), an effect that is blunted by aminooxyacetate (AOA), an inhibitor of transaminases (Figure 5F), as well as through GOT1 silencing (Figure 5G).

Consistent with a role for aspartate as a source of OAA to fuel MDH1-dependent NADH recycling in cells with mitochondrial dysfunction, we observed that aspartate supplementation increased NAD$^+$/NADH levels to a greater extent in mT80 cells, compared to mT7 and mT45 (Figure 5H), an increase that was due, at least in part, to flux through MDH1 (Figure 5I). Moreover, aspartate supplementation increased lactate secretion (Figure 5J) in an MDH1-dependent fashion (Figure 5K). Overall, these results indicate that cytosolic transamination of aspartate supports flux through MDH1 and synthesis of cytosolic malate, ultimately contributing to NADH recycling and glycolytic flux, to support proliferation of cells with mitochondrial dysfunction.

MDH1 Regulates Cell Migration

Finally, we investigated a functional consequence of the metabolic rewiring prompted by dysfunctional mitochondria. Recently, it was shown that production of ATP by glycolysis, rather than by mitochondrial oxidative phosphorylation (OXPHOS), supports cell migration (Yizhak et al., 2014). Of note, we found that the contribution of glycolysis to ATP production is higher in mT80 compared to mT7 and mT45 cells (Figure S7F). Therefore, we hypothesized that the switch to glycolytic ATP production might be associated with increased cell motility in our model. A proteome analysis of mT7 and mT80 cells, followed by gene ontology (GO) enrichment analysis, revealed that processes involved in cell migration and cytoskeleton remodeling were significantly altered between mT7 and mT80 cells (Figure 6A; Table S4). We therefore assessed cell migration in mT7, mT45, and mT80 cells by performing a wound-healing assay *in vitro*. We found that the migratory capacity increased proportionally to the degree of mitochondrial dysfunction, with mT80 cells displaying highest migration speed (Figures 6B and S7G). In line with this result, greater amounts of ATP were used for cytoskeletal remodeling in mT80, compared to mT7 and mT45 (Figure 6C). Finally, we assessed the role of MDH1 in supporting this process. We found that the migratory abilities of mT80 were markedly reduced upon silencing of *MDH1*, compared to non-targeting control (Figures 6D and S7H), and shMDH1 cells demonstrated reduced utilization of ATP for cytoskeleton dynamics (Figure 6E). In addition, we observed that co-localization of MDH1 with actin followed the trend of mitochondrial dysfunction in mT7, mT45, and mT80 cells (Figures 6F and 6G). Together, these results indicate that mitochondrial dysfunction is associated with increased migration and that MDH1 might play a role in this process by sustaining glycolytic ATP generation.

DISCUSSION

In this study, we exploited a panel of isogenic cell lines that harbor varying degrees of mtDNA mutation that affects ATP synthase, mTUNE, for investigating the effect of mitochondrial dysfunction on cell metabolism. Other studies have modeled mitochondrial dysfunction in isogenic cell lines. For instance, the expression of a dominant-negative mutant of polymerase gamma was used to induce the depletion of mtDNA in cultured cell lines, identifying links between mitochondrial function and epigenetic changes (Martínez-Reyes et al., 2016), and serine metabolism (Bao et al., 2016). The expression of a mutant variant of the mtDNA helicase Twinkle was used to model mitochondrial dysfunction, confirming the association between mitochondrial function and serine and purine biosynthetic pathways (Nikkanen et al., 2016). Compared to these models, the mTUNE model allows the generation of a panel of isogenic cell lines displaying varying degrees of mitochondrial function. In addition, mTUNE cells display mild mitochondrial dysfunction and might represent a more physiological model of mitochondrial function compared to pharmacological inhibition of respiration (Birsoy et al., 2015) or profound loss of mitochondrial respiratory complexes (Bao et al., 2016; Sullivan et al., 2015; Martínez-Reyes et al., 2016).

Reductive carboxylation is known to support proliferation of cancer cells with mitochondrial dysfunction (Mullen et al., 2011) or when treated with metformin (Liu et al., 2016), and contributes to *de novo* lipid synthesis under hypoxia (Metallo et al., 2011). Yet its biochemical determinants remain unclear. In this work, we demonstrate that reductive carboxylation supports metabolic flux through the NADH-consuming MDH1, regenerating cytosolic NADH to support glycolysis. Notably, the requirement of high NADH turnover to support glycolytic flux has been previously hypothesized, especially in conditions in which mitochondrial function is not sufficient to recycle cytosolic NADH and high biomass generation is required (Dai et al., 2016). For instance, high cytosolic NADH turnover has been suggested to support anabolic reactions that branch out of

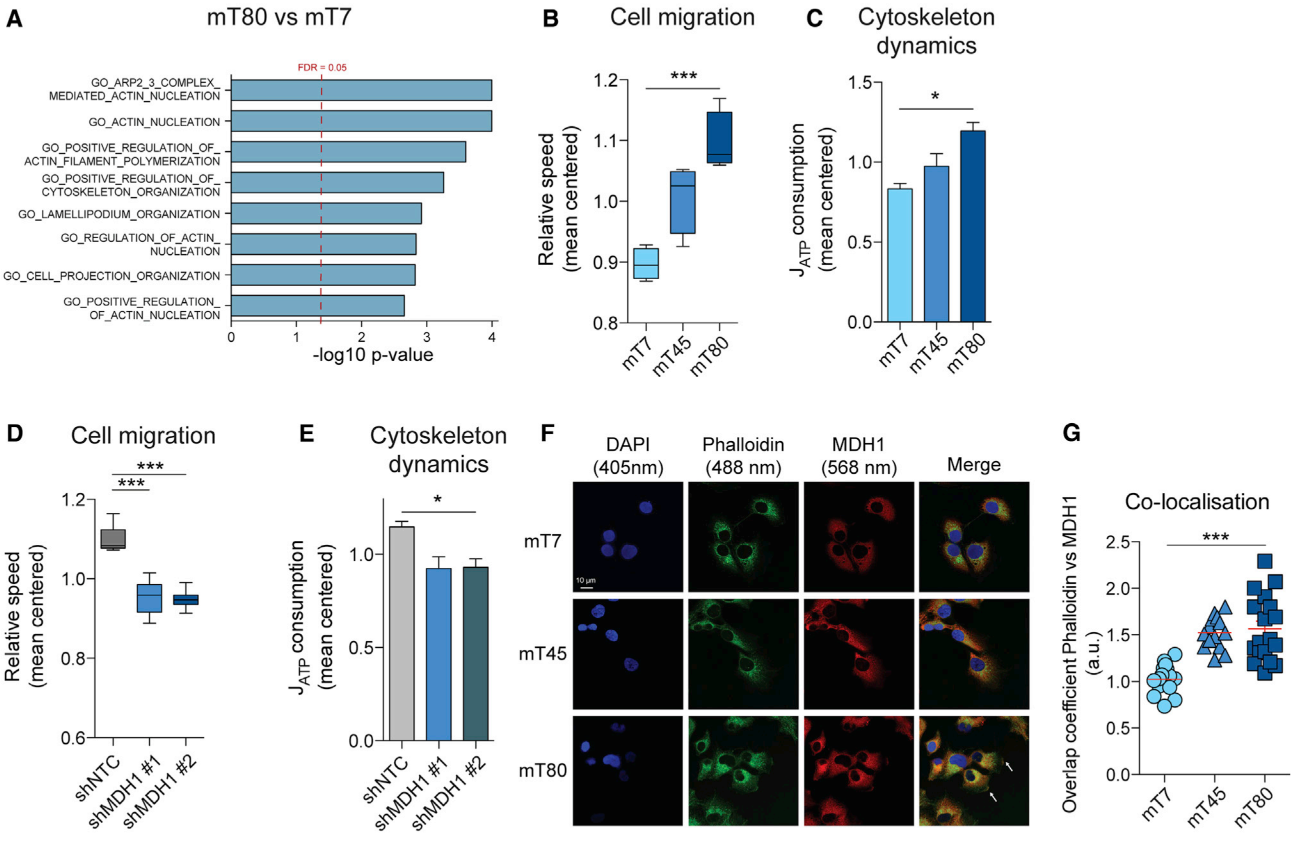

Figure 6. Mitochondrial Dysfunction Is Linked with Cell Migration

(A) Enrichment p values ($-\log_{10}$) of gene ontology (GO) biological processes involved in cell migration and cytoskeleton remodeling as obtained with measurements of protein abundance by proteomics. Red dashed line indicates false discovery rate (FDR) = 0.05.

(B and D) Migration speed of mT7, mT45, and mT80 cells (B) or shMDH1 mT80 cells (D) measured by wound healing assay.

(C and E) Values of J_{ATP} consumption due to cytoskeleton remodeling based on calculations from OCR and ECAR data upon treatment with 1 μM nocodazole in mT7, mT45, and mT80 cells (C) or mT80 shMDH1 cells (E).

(F) Immunofluorescence images of mT7, mT45, and mT80 cells stained with DAPI (blue), phalloidin (green), or antibody against MDH1 (red). White arrows indicate areas of co-localization between MDH1 and actin in mT80 cells.

(G) Quantification of co-localization between MDH1 and phalloidin (actin). Data were obtained from 20–30 ROIs per condition.

(B–E) Data are mean ± SEM from three to four independent cultures and were normalized on mean values of each experiment.

$^{*}p \leq 0.05$ and $^{***}p \leq 0.001$, ANOVA (B, C, and G) or Dunnett's test (D).

glycolysis, such as the serine biosynthesis pathway (Liberti and Locasale, 2016). In this scenario, cytosolic reductive carboxylation operates as substitute for MAS, tightly coupling the oxidation of glutamine with glycolysis and likely enabling glycolytic flux for ATP synthesis and biomass generation.

Our studies show that MDH1 is an important enzyme when high glycolytic capacity is required to support anabolic demands or to compensate for NAD redox imbalance upon mitochondrial dysfunction (see Figure 7 for a schematic). These results are in line with a recent study that demonstrated that MDH1 can recycle glycolytic NADH and support proliferation of cancer cells and activated lymphocytes (Hanse et al., 2017), two settings characterized by diminished mitochondrial function and high reliance on glycolysis (Vander Heiden et al., 2009; Pearce et al., 2013). Furthermore, our study concurs with the finding that MDH1, but not MDH2, is required for proliferation of lung cancer cell lines (Zhang et al., 2017). While the study from Hanse and colleagues (Hanse et al., 2017) lacked a formal demonstration of the role of reductive carboxylation and MDH1 in recycling NADH to fuel glycolysis, our functional and metabolic labeling studies provide compelling evidence that NADH shuttling couples cytosolic reductive carboxylation of glutamine with glycolysis in cells with mitochondrial dysfunction.

Recent studies have demonstrated that cells with mitochondrial dysfunction, through either genetic or pharmacological inhibition of the RC, experience a drop in intracellular aspartate and undergo a profound rewiring of the MAS (Birsoy et al., 2015; Sullivan et al., 2015). In this scenario, the reversal of MDH1 consumes NAD^+ and provides OAA for the generation of aspartate and nucleotide synthesis. Although our findings are in line with the depletion of aspartate in cells with mitochondrial dysfunction, we found increased flux through the forward, NAD^+-producing MDH1 reaction. Discrepancies between our findings and prior observations are likely due to the different level of

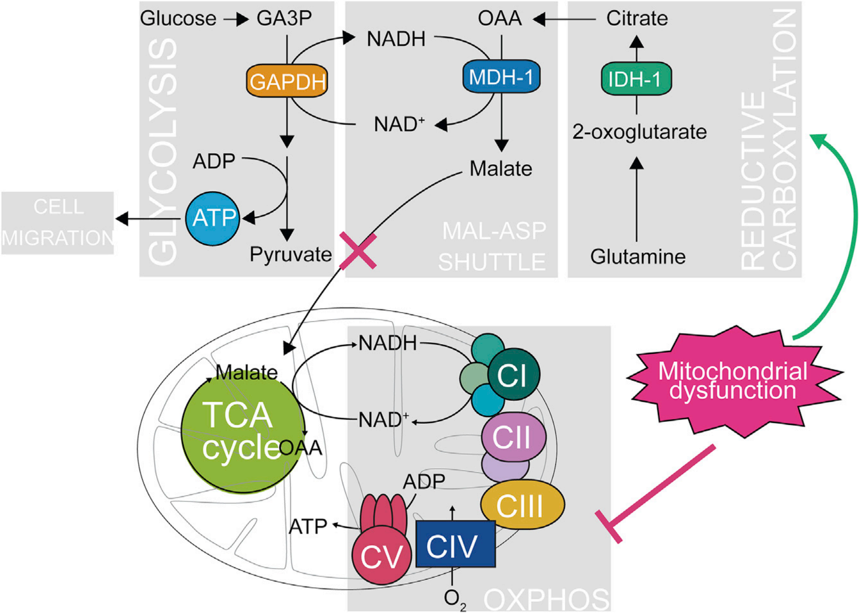

Figure 7. Reductive Glutamine Carboxylation Regulates NAD Redox Balance and Supports Glycolysis in Response to Mitochondrial Dysfunction

Reduced turnover of NADH by mitochondria leads to impairment of the MAS and increase of cytosolic NADH. This in turn induces reductive carboxylation of glutamine, providing carbons for NADH-coupled MDH1, thus regulating NAD redox state and enhancing GAPDH activity. Increased glycolytic turnover supports ATP production in the cytosol, and this is associated with cell migration.

mitochondrial dysfunction in these models. Indeed, mitochondrial dysfunction in mTUNE is milder than that triggered by phenformin (Birsoy et al., 2015) or by loss of CYTB (Sullivan et al., 2015) and might represent a more physiological dysregulation of mitochondrial function in which the MAS still operates in the forward direction.

We also provided evidence that MDH1 may interact with the key glycolytic enzyme GAPDH, enhancing the recycling of glycolytic NADH. Interaction of glycolytic enzymes into a single multienzyme complex has been observed previously (Menard et al., 2014). Importantly, multienzyme complexes can offer several advantages, including higher solvation and substrate channeling. Formation of a multienzyme complex between GAPDH and LDH has already been reported (Svedružić and Spivey, 2006), and NADH channeling between these two enzymes is known to be rate limiting for glycolytic flux. Moreover, the generation of a multienzyme complex for maximization of GAPDH flux is in line with the central role of GAPDH as the limiting step regulating aerobic glycolysis (Shestov et al., 2014). Our data suggest that the interaction between MDH1 and GAPDH might be dictated by mitochondrially driven changes in the biochemical environment of the cytosol, such as the availability of NAD^+ or pH. However, we do want to emphasize that our work has not conclusively demonstrated or characterized the physical interaction between GAPDH and MDH, and that more work is required to establish the biophysical and biochemical basis of this interaction.

Recently, published work has highlighted the link between mitochondrial dysfunction and acquisition of cell migratory abilities, whereby pharmacological inhibition of the RC (Porporato et al., 2014), genetic impairment of the TCA cycle (Loriot et al., 2012; Sciacovelli et al., 2016), or loss of transcriptional regulation for mitochondrial biogenesis (Torrano et al., 2016) can lead to acquisition of migratory properties and induce metastasis of cancer cells. In addition, we have recently reported that downregulation of nuclear encoded mitochondrial enzymes is associated with the induction of epithelial-to-mesenchymal transition (EMT), a genetic signature supporting migration and metastasis (Gaude and Frezza, 2016). Yet the underlying mechanisms remain unclear. Our results show that mitochondrial dysfunction is associated with increased production of ATP from glycolysis and increased migratory capacity. These results are in line with the observation that increased glycolytic versus oxidative generation of ATP increases cell migration (Yizhak et al., 2014) and with the recent evidence that glycolytic enzymes can sustain cell motility by localizing with components of the cytoskeleton and providing local generation of ATP (De Bock et al., 2013). Together, our results suggest that, in the presence of mitochondrial dysfunction, increased flux through MDH1 can enhance ATP yield from glycolysis and is associated with increased cell migration.

STAR★METHODS

Detailed methods are provided in the online version of this paper and include the following:

- KEY RESOURCES TABLE
- CONTACT FOR REAGENT AND RESOURCE SHARING
- EXPERIMENTAL MODEL AND SUBJECT DETAILS
- METHOD DETAILS
  - Cell growth assays
  - Oxygen consumption and Extracellular acidification rate measurement
  - Western blotting
  - Immunoprecipitation assay
  - Immunofluorescence assay
  - Proteomics
  - Quantification of m.8993 heteroplasmy
  - Fluorescence associated cell sorting (FACS)
  - NADH measurements
  - Metabolomics analysis
  - Metabolic modeling
  - Lentiviral vectors generation and transduction
  - Cell migration
  - qPCR

- QUANTIFICATION AND STATISTICAL ANALYSIS
- DATA AND SOFTWARE AVAILABILITY

SUPPLEMENTAL INFORMATION

Supplemental Information includes seven figures and four tables and can be found with this article online at https://doi.org/10.1016/j.molcel.2018.01.034.

ACKNOWLEDGMENTS

The work of E.G., C.S., and C.F. is supported by the Medical Research Council (MRC) Core Award to the MRC Cancer Unit (MC_UU_12022/7). E.G. thanks the MRC doctorate training (DTA studentship) for financial support. E.G. and C.F. thank Ana S.H. Costa for extensive support with mass spectrometry metabolomics. The work of A.D. was supported by the Q3 European Union Horizon 2020 grant SyMBioSys MSCA-ITN-2015-ETN #675585. Work of M.M. and P.A.G. was funded by the Medical Research Council, UK (MC_U105697135). T.B. and G.S. were funded by the Biochemical and Biophysical Research Council (grants BB/L020874/1 and BB/P018726/1) and the Wellcome Trust (097815/Z/11/Z). T.B. and G.S. thank Michael Duchen for helpful discussion. J.S.O. and S.P.C. were supported by the Medical Research Council (MC_UP_1201/4) and the Wellcome Trust (093734/Z/10/Z). We thank the laboratory of Costas Lyssiotis for sharing the constructs for targeting GOT1 and the laboratory of Eyal Gottlieb for sharing the *Sdhb*-deficient cells.

AUTHOR CONTRIBUTIONS

E.G. and C.F. designed the experiments and wrote the manuscript. E.G. performed all experiments and computer simulations. C.S. helped with immunoprecipitation analyses. G.S. and T.B. supervised the measurements of NAD(P)H autofluorescence experiments and helped with the interpretation of results. M.M. and P.A.G. provided cell lines, performed RFLP (restriction fragment length polymorphism) experiments, and helped with interpretation of results. J.S.O. and S.P.C. performed proteomics analysis. A.D. and J.S.-R. performed statistical analysis of proteomics data. All the authors read and commented on the manuscript. C.F. directed the research.

DECLARATION OF INTERESTS

The authors declare no competing interests.

Received: September 5, 2017
Revised: December 12, 2017
Accepted: January 25, 2018
Published: February 15, 2018

REFERENCES

Bao, X.R., Ong, S.-E., Goldberger, O., Peng, J., Sharma, R., Thompson, D.A., Vafai, S.B., Cox, A.G., Marutani, E., Ichinose, F., et al. (2016). Mitochondrial dysfunction remodels one-carbon metabolism in human cells. eLife *5*, e10575.

Birsoy, K., Wang, T., Chen, W.W., Freinkman, E., Abu-Remaileh, M., and Sabatini, D.M. (2015). An essential role of the mitochondrial electron transport chain in cell proliferation is to enable aspartate synthesis. Cell *162*, 540–551.

Blacker, T.S., and Duchen, M.R. (2016). Investigating mitochondrial redox state using NADH and NADPH autofluorescence. Free Radic. Biol. Med. *100*, 53–65.

Cairns, R.A., Harris, I.S., and Mak, T.W. (2011). Regulation of cancer cell metabolism. Nat. Rev. Cancer *11*, 85–95.

Cannino, G., El-Khoury, R., Pirinen, M., Hutz, B., Rustin, P., Jacobs, H.T., and Dufour, E. (2012). Glucose modulates respiratory complex I activity in response to acute mitochondrial dysfunction. J. Biol. Chem. *287*, 38729–38740.

Cardaci, S., Zheng, L., MacKay, G., van den Broek, N.J.F., MacKenzie, E.D., Nixon, C., Stevenson, D., Tumanov, S., Bulusu, V., Kamphorst, J.J., et al. (2015). Pyruvate carboxylation enables growth of SDH-deficient cells by supporting aspartate biosynthesis. Nat. Cell Biol. *17*, 1317–1326.

Dai, Z., Shestov, A.A., Lai, L., and Locasale, J.W. (2016). A flux balance of glucose metabolism clarifies the requirements of the Warburg effect. Biophys. J. *111*, 1088–1100.

De Bock, K., Georgiadou, M., Schoors, S., Kuchnio, A., Wong, B.W., Cantelmo, A.R., Quaegebeur, A., Ghesquière, B., Cauwenberghs, S., Eelen, G., et al. (2013). Role of PFKFB3-driven glycolysis in vessel sprouting. Cell *154*, 651–663.

Dickman, K.G., and Mandel, L.J. (1990). Differential effects of respiratory inhibitors on glycolysis in proximal tubules. Am. J. Physiol. *258*, F1608–F1615.

Fendt, S.-M., Bell, E.L., Keibler, M.A., Olenchock, B.A., Mayers, J.R., Wasylenko, T.M., Vokes, N.I., Guarente, L., Vander Heiden, M.G., and Stephanopoulos, G. (2013). Reductive glutamine metabolism is a function of the α-ketoglutarate to citrate ratio in cells. Nat. Commun. *4*, 2236.

Frezza, C., Zheng, L., Tennant, D.A., Papkovsky, D.B., Hedley, B.A., Kalna, G., Watson, D.G., and Gottlieb, E. (2011). Metabolic profiling of hypoxic cells revealed a catabolic signature required for cell survival. PLoS ONE *6*, e24411.

Gammage, P.A., Rorbach, J., Vincent, A.I., Rebar, E.J., and Minczuk, M. (2014). Mitochondrially targeted ZFNs for selective degradation of pathogenic mitochondrial genomes bearing large-scale deletions or point mutations. EMBO Mol. Med. *6*, 458–466.

Gammage, P.A., Gaude, E., Van Haute, L., Rebelo-Guiomar, P., Jackson, C.B., Rorbach, J., Pekalski, M.L., Robinson, A.J., Charpentier, M., Concordet, J.-P., et al. (2016a). Near-complete elimination of mutant mtDNA by iterative or dynamic dose-controlled treatment with mtZFNs. Nucleic Acids Res. *44*, 7804–7816.

Gammage, P.A., Van Haute, L., and Minczuk, M. (2016b). Engineered mtZFNs for manipulation of human mitochondrial DNA heteroplasmy. Methods Mol. Biol. *1351*, 145–162.

Gaude, E., and Frezza, C. (2016). Tissue-specific and convergent metabolic transformation of cancer correlates with metastatic potential and patient survival. Nat. Commun. *7*, 13041.

Gaude, E., Chignola, F., Spiliotopoulos, D., Spitaleri, A., Ghitti, M.M., Garcia-Manteiga, J., Mari, S., and Musco, G. (2013). muma, an R package for metabolomics univariate and multivariate statistical analysis. Curr. Metabolomics *1*, 180–189.

Hanse, E.A., Ruan, C., Kachman, M., Wang, D., Lowman, X.H., and Kelekar, A. (2017). Cytosolic malate dehydrogenase activity helps support glycolysis in actively proliferating cells and cancer. Oncogene *36*, 3915–3924.

Jiang, L., Shestov, A.A., Swain, P., Yang, C., Parker, S.J., Wang, Q.A., Terada, L.S., Adams, N.D., McCabe, M.T., Pietrak, B., et al. (2016). Reductive carboxylation supports redox homeostasis during anchorage-independent growth. Nature *532*, 255–258.

King, M.P., and Attardi, G. (1989). Human cells lacking mtDNA: repopulation with exogenous mitochondria by complementation. Science *246*, 500–503.

Lewis, C.A., Parker, S.J., Fiske, B.P., McCloskey, D., Gui, D.Y., Green, C.R., Vokes, N.I., Feist, A.M., Vander Heiden, M.G., and Metallo, C.M. (2014). Tracing compartmentalized NADPH metabolism in the cytosol and mitochondria of mammalian cells. Mol. Cell *55*, 253–263.

Liberti, M.V., and Locasale, J.W. (2016). The Warburg effect: how does it benefit cancer cells? Trends Biochem. Sci. *41*, 211–218.

Liu, X., Romero, I.L., Litchfield, L.M., Lengyel, E., and Locasale, J.W. (2016). Metformin targets central carbon metabolism and reveals mitochondrial requirements in human cancers. Cell Metab. *24*, 728–739.

Loriot, C., Burnichon, N., Gadessaud, N., Vescovo, L., Amar, L., Libé, R., Bertherat, J., Plouin, P.-F., Jeunemaitre, X., Gimenez-Roqueplo, A.-P., and Favier, J. (2012). Epithelial to mesenchymal transition is activated in metastatic pheochromocytomas and paragangliomas caused by SDHB gene mutations. J. Clin. Endocrinol. Metab. *97*, E954–E962.

Mackay, G.M., Zheng, L., van den Broek, N.J.F., and Gottlieb, E. (2015). Analysis of cell metabolism using LC-MS and isotope tracers. Methods Enzymol. *561*, 171–196.

Mahadevan, R., and Schilling, C.H. (2003). The effects of alternate optimal solutions in constraint-based genome-scale metabolic models. Metab. Eng. *5*, 264–276.

Marroquin, L.D., Hynes, J., Dykens, J.A., Jamieson, J.D., and Will, Y. (2007). Circumventing the Crabtree effect: replacing media glucose with galactose increases susceptibility of HepG2 cells to mitochondrial toxicants. Toxicol. Sci. *97*, 539–547.

Martínez-Reyes, I., Diebold, L.P., Kong, H., Schieber, M., Huang, H., Hensley, C.T., Mehta, M.M., Wang, T., Santos, J.H., Woychik, R., et al. (2016). TCA cycle and mitochondrial membrane potential are necessary for diverse biological functions. Mol. Cell *61*, 199–209.

Menard, L., Maughan, D., and Vigoreaux, J. (2014). The structural and functional coordination of glycolytic enzymes in muscle: evidence of a metabolon? Biology (Basel) *3*, 623–644.

Metallo, C.M., Gameiro, P.A., Bell, E.L., Mattaini, K.R., Yang, J., Hiller, K., Jewell, C.M., Johnson, Z.R., Irvine, D.J., Guarente, L., et al. (2011). Reductive glutamine metabolism by IDH1 mediates lipogenesis under hypoxia. Nature *481*, 380–384.

Mookerjee, S.A., Gerencser, A.A., Nicholls, D.G., and Brand, M.D. (2017). Quantifying intracellular rates of glycolytic and oxidative ATP production and consumption using extracellular flux measurements. J. Biol. Chem. *292*, 7189–7207.

Mullen, A.R., Wheaton, W.W., Jin, E.S., Chen, P.-H., Sullivan, L.B., Cheng, T., Yang, Y., Linehan, W.M., Chandel, N.S., and DeBerardinis, R.J. (2011). Reductive carboxylation supports growth in tumour cells with defective mitochondria. Nature *481*, 385–388.

Nikkanen, J., Forsström, S., Euro, L., Paetau, I., Kohnz, R.A., Wang, L., Chilov, D., Viinamäki, J., Roivainen, A., Marjamäki, P., et al. (2016). Mitochondrial DNA replication defects disturb cellular dNTP pools and remodel one-carbon metabolism. Cell Metab. *23*, 635–648.

Orth, J.D., Thiele, I., and Palsson, B.Ø. (2010). What is flux balance analysis? Nat. Biotechnol. *28*, 245–248.

Pavlova, N.N., and Thompson, C.B. (2016). The emerging hallmarks of cancer metabolism. Cell Metab. *23*, 27–47.

Pearce, E.L., Poffenberger, M.C., Chang, C.-H., and Jones, R.G. (2013). Fueling immunity: insights into metabolism and lymphocyte function. Science *342*, 1242454.

Porporato, P.E., Payen, V.L., Pérez-Escuredo, J., De Saedeleer, C.J., Danhier, P., Copetti, T., Dhup, S., Tardy, M., Vazeille, T., Bouzin, C., et al. (2014). A mitochondrial switch promotes tumor metastasis. Cell Rep. *8*, 754–766.

Porteous, W.K., James, A.M., Sheard, P.W., Porteous, C.M., Packer, M.A., Hyslop, S.J., Melton, J.V., Pang, C.Y., Wei, Y.H., and Murphy, M.P. (1998). Bioenergetic consequences of accumulating the common 4977-bp mitochondrial DNA deletion. Eur. J. Biochem. *257*, 192–201.

Ritchie, M.E., Phipson, B., Wu, D., Hu, Y., Law, C.W., Shi, W., and Smyth, G.K. (2015). limma powers differential expression analyses for RNA-sequencing and microarray studies. Nucleic Acids Res. *43*, e47.

Ros, S., and Schulze, A. (2013). Balancing glycolytic flux: the role of 6-phosphofructo-2-kinase/fructose 2,6-bisphosphatases in cancer metabolism. Cancer Metab. *1*, 8.

Salabei, J.K., Gibb, A.A., and Hill, B.G. (2014). Comprehensive measurement of respiratory activity in permeabilized cells using extracellular flux analysis. Nat. Protoc. *9*, 421–438.

Sciacovelli, M., Gaude, E., Hilvo, M., and Frezza, C. (2014). The metabolic alterations of cancer cells. Methods Enzymol. *542*, 1–23.

Sciacovelli, M., Gonçalves, E., Johnson, T.I., Zecchini, V.R., da Costa, A.S.H., Gaude, E., Drubbel, A.V., Theobald, S.J., Abbo, S.R., Tran, M.G.B., et al. (2016). Fumarate is an epigenetic modifier that elicits epithelial-to-mesenchymal transition. Nature *537*, 544–547.

Shestov, A.A., Liu, X., Ser, Z., Cluntun, A.A., Hung, Y.P., Huang, L., Kim, D., Le, A., Yellen, G., Albeck, J.G., and Locasale, J.W. (2014). Quantitative determinants of aerobic glycolysis identify flux through the enzyme GAPDH as a limiting step. eLife *3*, e03342.

Son, J., Lyssiotis, C.A., Ying, H., Wang, X., Hua, S., Ligorio, M., Perera, R.M., Ferrone, C.R., Mullarky, E., Shyh-Chang, N., et al. (2013). Glutamine supports pancreatic cancer growth through a KRAS-regulated metabolic pathway. Nature *496*, 101–105.

Sullivan, L.B., Gui, D.Y., Hosios, A.M., Bush, L.N., Freinkman, E., and Vander Heiden, M.G. (2015). Supporting aspartate biosynthesis is an essential function of respiration in proliferating cells. Cell *162*, 552–563.

Svedruzić, Ž.M., and Spivey, H.O. (2006). Interaction between mammalian glyceraldehyde-3-phosphate dehydrogenase and L-lactate dehydrogenase from heart and muscle. Proteins *63*, 501–511.

Torrano, V., Valcarcel-Jimenez, L., Cortazar, A.R., Liu, X., Urosevic, J., Castillo-Martin, M., Fernández-Ruiz, S., Morciano, G., Caro-Maldonado, A., Guiu, M., et al. (2016). The metabolic co-regulator PGC1α suppresses prostate cancer metastasis. Nat. Cell Biol. *18*, 645–656.

Vander Heiden, M.G., Cantley, L.C., and Thompson, C.B. (2009). Understanding the Warburg effect: the metabolic requirements of cell proliferation. Science *324*, 1029–1033.

Väremo, L., Nielsen, J., and Nookaew, I. (2013). Enriching the gene set analysis of genome-wide data by incorporating directionality of gene expression and combining statistical hypotheses and methods. Nucleic Acids Res. *41*, 4378–4391.

Yizhak, K., Le Dévédec, S.E., Rogkoti, V.M., Baenke, F., de Boer, V.C., Frezza, C., Schulze, A., van de Water, B., and Ruppin, E. (2014). A computational study of the Warburg effect identifies metabolic targets inhibiting cancer migration. Mol. Syst. Biol. *10*, 744.

Zhang, B., Tornmalm, J., Widengren, J., Vakifahmetoglu-Norberg, H., and Norberg, E. (2017). Characterization of the role of the malate dehydrogenases to lung tumor cell survival. J. Cancer *8*, 2088–2096.

Zieliński, Ł.P., Smith, A.C., Smith, A.G., and Robinson, A.J. (2016). Metabolic flexibility of mitochondrial respiratory chain disorders predicted by computer modelling. Mitochondrion *31*, 45–55.

STAR★METHODS

KEY RESOURCES TABLE

REAGENT or RESOURCE	SOURCE	IDENTIFIER
Antibodies		
mouse anti-human GAPDH	Abcam	ab8245; RRID: AB_2107448
mouse anti-human Mitochondria OXPHOS cocktail	Origene	MS601-360; RRID: AB_1619331
rabbit anti-human LDH	Abcam	ab47010; RRID: AB_1952042
rabbit anti-human MDH1	Abcam	ab180152; RRID: AB_1651605
rabbit anti-NDI-1	Cambridge Research Biochemicals	N/A
mouse anti-human MDH1	Abcam	ab76616; RRID: AB_1523905
mouse anti-human TOMM20	Abcam	ab56783; RRID: AB_945896
rabbit anti-human Calnexin	Abcam	ab22595; RRID: AB_2069006
Bacterial and Virus Strains		
DH5α Max Efficiency Competent cells	Thermo Fisher	18258012
Chemicals, Peptides, and Recombinant Proteins		
Rotenone	Sigma-Aldrich	R8875
Oligomycin	Sigma-Aldrich	O4876
FCCP	Sigma-Aldrich	C2920
Antimycin A	Sigma-Aldrich	A8674
Digitonin	Sigma-Aldrich	D141
TMPD	Sigma-Aldrich	T7394
Sodium azide	Sigma-Aldrich	S2002
Nocodazole	Sigma-Aldrich	M1404
Heptelidic acid	Abcam	Ab144269
Amino-oxiacetate	Sigma-Aldrich	C13408
Duroquinol	TCI America	T0822
Critical Commercial Assays		
Dynabeads M-270 Epoxy beads	Life Technology	14311D
Deposited Data		
Raw images	This paper; and Mendeley data	http://dx.doi.org/10.17632/bmvrzxgs6c.1
Experimental Models: Cell Lines		
mTUNE cells	(Gammage et al., 2016a)	N/A
SDH-null cells	(Cardaci et al., 2015)	N/A
Oligonucleotides		
Primers for human IDH1 FWD GTGTGCAAAATCTTCAATTGACTT	This paper	N/A
Primers for human IDH1 REV GGTGACATACCTGGTACATAACTTTG	This paper	N/A
Primers for human IDH2 FWD GGAGCCCGAGGTCAAAATAC	This paper	N/A
Primers for human IDH2 REV TGGCAGTTCATCAAGGAGAA	This paper	N/A
Primers for human GOT1 FWD CAACTGGGATTGACCCAACT	This paper	N/A
Primers for human GOT1 REV GGAACAGAAACCGGTGCTT	This paper	N/A
Primers for human actin	QIAGEN	QT00095431
Recombinant DNA		
pTRIPZ-shMDH1	Dharmacon	V2THS_211486
pTRIPZ-shMDH1	Dharmacon	V3THS_324429
pTRIPZ-shIDH1	Dharmacon	V2THS_217815

(*Continued on next page*)

Continued

REAGENT or RESOURCE	SOURCE	IDENTIFIER
pTRIPZ-shIDH1	Dharmacon	V3THS_320100
pTRIPZ-shIDH2	Dharmacon	V2THS_238370
pTRIPZ-shIDH2	Dharmacon	V3THS_412536
pWPI-NDI1	(Cannino et al., 2012)	N/A
pLKO-TetON-shGOT1	(Son et al., 2013)	TRCN0000220880
pLKO-TetON-shGOT1	(Son et al., 2013)	TRCN0000034784
Software and Algorithms		
Muma R package	(Gaude et al., 2013)	https://cran.r-project.org/web/packages/muma/index.html
Graphpad v5.0a	Prism	N/A

CONTACT FOR REAGENT AND RESOURCE SHARING

Further information and requests for resources and reagents should be directed to and will be fulfilled by the Lead Contact, Christian Frezza (cf366@mrc-cu.cam.ac.uk).

EXPERIMENTAL MODEL AND SUBJECT DETAILS

mTUNE cells were generated by Dr Michal Minczuk's lab and derive from female human osteosarcoma 143B (RRID: CVCL_2270) cybrid cells (Porteous et al., 1998), after correction of m.8993T>G mutation with mitochondrially-targeted zinc finger nucleases (Gammage et al., 2016a). Authentication was performed by assessing m.8993T>G heteroplasmy (detailed below). Except when indicated differently, cells were cultured in Dulbecco's modified Eagle's medium (DMEM, Life Technology cat. no. 41966-029) containing 25 mM glucose and 4 mM glutamine, with added 10% v/v fetal bovine serum (FBS) and grown in a humidified incubator at 37°C and 5% CO_2. Cells were allowed to grow for two to three days until 90%–95% confluent before next subpassaging.

METHOD DETAILS

Cell growth assays

For cell proliferation assays $2*10^4$ cells were seeded in 24-well plates and allowed to attach for at least 16 hours. Medium was then changed to normal DMEM or DMEM with added/substituted nutrients or drugs. Cell growth in galactose was performed by culturing cells in glucose-free and pyruvate-free DMEM (Life Technology cat. no. 11966-025), with added 10% v/v FBS, 25 mM D-galactose (Sigma, G0750) and 1 mM sodium pyruvate (Sigma, P2256). To assess cell proliferation in the presence of aminooxiacetate (AOA) and aspartate normal DMEM was supplemented with varying concentrations of AOA (Sigma-Aldrich, C13408) and 4 mM L-aspartic acid (Sigma-Aldrich, A9256). At least 4 independent replicates were recorded for each condition, in each experiment. Cell growth was assessed with an IncuCyte FLR (Essen Bioscience) and assay was stopped when all cell conditions reached full confluency or confluency started to decrease consistently.

Oxygen consumption and Extracellular acidification rate measurement

To assess oxygen consumption rate (OCR) and extracellular acidification rate (ECAR) $6*10^4$ cells were seeded the night before experiment in XF^e24 Cell Culture microplate in 100 μL normal DMEM. The next day cells were washed twice in phosphate buffer saline (PBS) and medium was replaced with 675μL of bicarbonate-free DMEM (Sigma-Aldrich, D5030) supplemented with 25 mM glucose, 1 mM pyruvate, 4 mM glutamine, 40 μM phenol red and 1% v/v FBS. To eliminate residues of carbonic acid from medium, cells were incubated for at least 30 minutes at 37°C with atmospheric CO_2 in a non-humidified incubator. OCR and ECAR were assayed in a Seahorse XF-24 extracellular flux analyzer by the addition via ports A–C of 1 μM oligomycin (port A), 1 μM carbonyl cyanide-p-trifluoromethoxyphenylhydrazone (FCCP, port B), 1 μM rotenone and 1 μM antimycin A (port C). Two or three measurement cycles of 2-min mix, 2-min wait, and 4-min measure were carried out at basal condition and after each injection. At the end of the experiment, each well was washed twice with 1mL of PBS and proteins were extracted with 100μL of radioimmune precipitation assay (RIPA) lysis medium (150 mM NaCl, 50 mM Tris, 1 mM EGTA, 1 mM EDTA, 1% (v/v) Triton X-100, 0.5% (w/v) sodium deoxycholate, 0.1% (v/v) SDS, pH 7.4) at room temperature. Plates were incubated at −80°C for 30 min and allowed to thaw at room temperature. Protein concentration in each well was measured by a BCA assay according to the manufacturer's instructions (Thermo). OCR and ECAR values were normalized on total μg of proteins in each well.

Activity of individual respiratory complexes was assessed by following a modified version of the method proposed by Salabei and colleagues (Salabei et al., 2014). Cells were seeded in XF^e24 Cell Culture microplate as mentioned above. On the day of experiment

each well was washed twice with 500μL of mannitol and sucrose buffer (70 mM sucrose, 220 mM mannitol, 10 mM KH_2PO_4, 5 mM $MgCl_2$, 2 mM HEPES, 1 mM EGTA, 4 mg/mL fatty acid-free bovine serum albumin, pH 7.2), replaced with 675μL of MAS buffer with added 20μg/mL digitonin (Sigma-Aldrich, cat. no. D141) and plate was immediately inserted into the Seahorse XF-24 analyzer. Activity of complex I and II was assayed on the same plate by adding in port A-D 5 mM glutamate and 2.5 mM malate (port A), 1 μM rotenone (port B), 10 mM succinate (port C) and 1μM antimicyn A (port D). Activity of complex III was assayed by addition via port A-B of 500 μM duroquinol (port A) and 1 μM Antimycin A (port B). Activity of complex IV was assessed by adding in port A-B 500μM TMPD and 2 mM ascorbate (port A) and 20 mM sodium azide (port B). All drug solutions were prepared in MAS buffer. Two or three measurement cycles of 2-min mix, 2-min wait, and 3-min measure were carried out at basal condition and after each injection. Protein concentrations in each well were determined as detailed above. OCR measurements were normalized on total μg of proteins in each well.

Estimation of ATP production and consumption from oxidative phosphorylation and glycolysis was obtained as described by Mookerjee et al. (2017). Briefly, $4*10^4$ cells were seeded in XF^e24 Cell Culture microplate in 100 μL normal DMEM. The next day cells were washed twice with, and medium was replaced by, 675 μL Krebs-Ringer phosphate HEPES (KRPH) medium (2 mM HEPES, 136 mM NaCl, 2 mM NaH_2PO_4, 3.7 mM KCl, 1 mM $MgCl_2$, 1.5 mM $CaCl_2$, 0.1% (w/v) fatty-acid-free bovine serum albumin, pH 7.4 at 37°C) and incubated for 30 minutes at 37°C with atmospheric CO_2. OCR and ECAR were assayed in a Seahorse XF-24 extracellular flux analyzer by the addition via ports A–D of 10mM glucose (port A), 1 μM oligomycin (port B), 1 μM carbonyl cyanide-p-trifluoromethoxyphenylhydrazone (FCCP, port C), 1 μM rotenone and 1 μM antimycin A (port D). To assess ATP consumption by cytoskeleton dynamics injections were: (A) 10 mM glucose, (B) 1 μM nocodazole (Sigma-Aldrich cat. no. M1404), (C) 1 μM oligomycin and (D) 1 μM rotenone and 1 μM antimycin A. Two or three measurement cycles of 1-min mix, 1-min wait, and 3-min measure were carried out at basal condition and after each injection. At the end of the experiment, protein concentration was quantified as described above. Calculations of J_{ATP} were performed as described in Mookerjee et al. (2017). For each experiment, 5-7 technical replicates were collected per condition.

Western blotting

For analysis of protein expression proteins were extracted in RIPA lysis medium (150 mM NaCl, 50 mM Tris, 1 mM EGTA, 1 mM EDTA, 1% (v/v) Triton X-100, 0.5% (w/v) sodium deoxycholate, 0.1% (v/v) SDS, pH 7.4) at room temperature. 20-50μg of protein was heated at 70°C for 10min in the presence of sample buffer 1 × (Bolt loading buffer 1 × (Life Technologies cat. no. B0007) supplemented with 4% β-mercaptoethanol (Sigma-Aldrich cat. no. M6250)). Samples were then loaded onto Bolt gel 4%–12% Bis-Tris (Invitrogen cat. no. NW04122BOX) and run using MES 1 × buffer (Life Technologies cat. no. B0002) at 200V constant for 30-40min. Dry transfer of the gels was carried out using IBLOT2 system (Life Technologies). Membranes were then incubated in blocking buffer (5% milk in TBS 1 × + 0.01% Tween 20) for 30 minutes at room temperature. Primary antibodies in blocking buffer were incubated overnight at 4°C or 2 hours at room temperature. Secondary antibodies (conjugated with 680 or 800nm fluorophores from Li-Cor) were diluted 1:2,000 in blocking buffer and incubated for 1h at room temperature. Images were acquired using a Li-Cor Odyssey CLx system linked with Image Studio 5.2 software (Li-Cor). Primary antibodies were: mouse anti-human GAPDH (Abcam cat. no. ab8245), mouse anti-human Mitochondria OXPHOS cocktail (Origene cat. no. MS601-360), rabbit anti-human LDH (Abcam cat. no. ab47010), rabbit anti-human MDH1 (Abcam cat. no. ab180152), rabbit anti-human Calnexin (Abcam cat. no. ab22595), rabbit anti-NDI-1 (Cambridge Research Biochemicals), mouse anti-human TOMM20 (Abcam cat. no. ab56783).

Immunoprecipitation assay

For immunoprecipitation assay cells were seeded in 15 cm dishes and allowed to grow until 95% confluent. Cells were then washed twice with PBS on ice, 500μL of lysis buffer (140 mM NaCl, 5mM EDTA, 1% Triton X-100, 20 mM Tris pH 7.4) was added and cells were scraped and collected. Extracted samples were incubated overnight at −20°C, centrifuged at 16000 g for 2 minutes at 4°C and supernatant was collected. For each immunoprecipitation reaction, 35 μg of mouse anti-human GAPDH (Abcam cat. no. ab8245) or mouse anti-human MDH1 (Abcam cat. no. ab76616) were coupled to 1.5 mg Dynabeads M-270 Epoxy beads (Life Technologies, cat. no. 14311D) following manufacturer's instructions. Antibody-coupled beads were incubated with 3 mg of protein lysate per condition (total volume 1 mL) on a spinning wheel for 30 minutes at 4°C. After incubation, samples were placed on a magnet rack and beads were washed three times with lysis buffer. Elution was performed by two cycles of beads resuspension in 20 μL of sample buffer 1 × (see above) followed by incubation at 70°C for 10 minutes. Immunoprecipitation and co-immunoprecipitation were assayed via western blotting.

Immunofluorescence assay

$2*10^4$ cells were seeded in 8-well μ-Slide chambers (Ibidi Labware cat. no. 80821). The next day cells were washed twice with PBS, fixed with 4% formaldehyde for 10 minutes at room temperature and washed twice with tris-buffered saline (TBS, 50 mM Tris, 150 mM NaCl, pH 7.6). Cells were then permeabilised with 2% BSA, 0,1% Triton X-100 in TBST (TBS + 0,1% Tween 20) for 10 minutes at room temperature, washed three times with TBS and blocked with 1% BSA, 10% goat serum (Abcam) in TBST for 30 minutes at room temperature. Cells were washed three times with TBS and incubated overnight at 4°C with a solution containing mouse anti-human GAPDH (Abcam cat. no. ab8245) and rabbit anti-human MDH1 (Abcam cat. no. ab180152) at a 1:100 dilution. After incubation with primary antibodies, cells were washed three times with TBS (5 minutes each wash) and stained with goat anti-mouse IgG

coupled with Alexa Fluor 488 (Thermo Fisher Scientific cat. no. A11001) and goat anti-rabbit IgG coupled with Alexa Fluor 568 (Thermo Fisher Scientific cat. no. A11011) for 2 hours at room temperature in the dark. Cells were washed three times with TBS, DNA was stained with a solution of 1 μg/mL of diamidino 2-phenylindole (DAPI) or with a solution of DAPI supplemented with 1 μM Alexa Fluor Phalloidin 488 (Thermo Fisher cat. no. A12379) for ten minutes at room temperature and cells were washed three times with TBS before image acquisition. Images were acquired on Leica confocal microscope TCS SP5 with 63 × objective. Each channel was acquired separately to avoid bleed through and laser intensity, magnification, and microscope settings were maintained equal for all conditions. Co-localization of GAPDH and MDH1 or Actin and MDH1 was quantified by assessing the overlap coefficient between channels with the use of Volocity software v6.3 (PerkinElmer).

Proteomics

Cells were seeded in 15 cm dishes and allowed to grow until 95% confluent. Cells were then washed twice with PBS on ice and 500 μL of extraction buffer (20 mM HEPES, 8 M urea, protease inhibitors cocktail 1X (Sigma-Aldrich cat. no. P8340) and phosphatase inhibitors cocktail (Sigma-Aldrich cat. no. P2850 and P5726), pH 8) were added. Protein concentration was quantified as described above and 400 μg of proteins were submitted to further processing. Samples from three independent experiments were collected.

Protein samples (9 × 360 μg each) were prepared in 200 μL lysis buffer (8M urea, 20 mM HEPES pH8, supplemented with protease and phosphatase inhibitors) were reduced with 5 mM DTT at 56°C for 30 min and alkylated with 10 mM iodoacetamide in the dark at room temperature for 30 min. After this, the samples were digested with Lys-C (mass spectrometry grade, Promega), 120:1 (protein: Lys-C ratio, w/w) for 4.5hr at 25°C. Next, the samples were diluted from 8M to 1.8 M urea with 20 mM HEPES (pH 8.5) and were digested with trypsin (Promega) 45:1 (protein: trypsin ratio, w/w) over night, at 25°C. Digestion was stopped by the addition of trifluoroacetic acid (TFA) to a final concentration of 1%. Any precipitates were removed by centrifugation at 8000 rpm for 8 min. The supernatants were desalted using a home-made C18 stage tips (3M Empore) contained 7 mg of poros R3 (Applied Biosystems) resin. Bound peptides were eluted with 30%–80% acetonitrile (MeCN) in 0.1% TFA and lyophilized.

Peptide mixtures from each condition was re-suspended in 100 μL of 3% MeCN and the peptide concentrations were determined by Pierce Quantitative Colorimetric Peptide assay (Thermo Scientific) according to manufacturer instructions, except the absorbance was measured by NanoDrop Spectrophotometers (Thermo Scientific) at 480nm. TMT 10plex reagent (Thermo Fisher Scientific) of 0.8 mg each was re-constituted in 41 μL anhydrous MeCN. The labeling reaction was performed in 150 mM triethylammonium bicarbonate for 1hr at room temperature (r.t.), then terminated by incubation with 8 μL 5% hydroxylamine for 15 min. The labeled peptides were combined into a single sample and partially dried to remove acetonitrile in a SpeedVac. The labeled peptides mixture was desalted using Sep-Pak Plus Short tC18 cartridges (Waters). Bound peptides were eluted with 60% acetonitrile in 0.5% acetic acid and lyophilized.

About 100 μg of the labeled peptides were separated on an offline, high pressure liquid chromatography (HPLC). The experiment was carried out using XBridge BEH130 C18, 5 μm, 2.1 × 150mm (Waters) column with XBridge BEH C18 5 μm Van Guard cartridge, connected to an Ultimate 3000 Nano/Capillary LC System (Dionex). Peptides were separated with a gradient of 1%–90% B (A: 5% MeCN/10 mM ammonium bicarbonate, pH8; B: MeCN/10 mM ammonium bicarbonate, pH8, [9:1]) in 60 min at a flow rate of 250 μl/min. A total of 60 fractions were collected, they were combined into 20 fractions and partially dried in a Speed Vac to about 50 μL. The rest of the lyophilized labeled peptides were re-suspended in 1.5 mL of loading buffer (50% MeCN/ 2M lactic acid) and incubated with TiO_2 beads (12 mg, prewashed with loading buffer) for 1 hour at r.t. while shaking. After incubation, TiO_2 beads were transferred to a home-made C18 stage tips, washed on tip twice with loading buffer and once with 50% MeCN/ 0.1% TFA. Phosphopeptides were eluted sequentially with 50 mM K_2HPO_4, pH10; 50% MeCN/50mM K_2HPO_4, pH10 and 50% MeCN/0.1% TFA. The eluates were combined, acidified with formic acid, partially dried in a SpeedVac and desalted with home-made C18 stage tip (3M Empore) that contained 1 mg of Poros R3 resin (Applied Biosystems).

Liquid chromatography was performed on a fully automated Ultimate 3000 RSLC nano System (Thermo Scientific)) fitted with a 100 μm x 2 cm PepMap100 C18 nano trap column and a 75 μm × 25 cm reverse phase C18 nano column (Aclaim PepMap, Thermo Scientific). Samples were separated using a binary gradient consisting of buffer A (2% MeCN, 0.1% formic acid) and buffer B (80% MeCN, 0.1% formic acid). Peptides were dissolved in solvent A and eluted with a step gradient of 5 to 50% B in 87-105 min, 50 to 90% B in 6-10 min, with a flow rate of 300 nL/min. The HPLC system was coupled to a Q Exactive Plus mass spectrometer (Thermo Scientific) equipped with a nanospray ion source. The mass spectrometer was operated in standard data dependent mode, performed MS full-scan at 350-1600 m/z range, with a resolution of 140000. This was followed by MS2 acquisitions of the 15 most intense ions with a resolution of 35000 and NCE of 32%. MS target values of 3e6 and MS2 target values of 1e5 were used. Isolation window of precursor was set at 1.2 Da and dynamic exclusion of sequenced peptides was enabled for 40 s.

The acquired MSMS raw files were processed using MaxQuant (Cox and Mann) with the integrated Andromeda search engine (v.1.5.5.1). MSMS spectra were searched against *Homo sapiens*, UniProt Fasta database (Jan 2017). Carbamidomethylation of cysteines was set as fixed modification, while methionine oxidation, N-terminal acetylation (protein) (for both parameters groups) and phosphorylation (STY) (for phospho- group only) were set as variable modifications. Protein quantification requires 1 (unique+ razor) peptide. Other parameters in MaxQuant were set to default values. MaxQuant output file, proteinGroups.txt was then processed with Perseus software (v 1.5.5.0). After uploading the matrix, the data was filtered, to remove identifications from reverse database and modified peptide only, and common contaminants. Each peptide channel was normalized to the median and log2 transformed.

Quantification of m.8993 heteroplasmy

Heteroplasmy at position m.8993 was measured using a previously described PCR RFLP assay, exploiting the creation of a unique SmaI/XmaI site in the mutated molecule (Gammage et al., 2016b). Inclusion of [α 32 P]-dCTP in a final cycle of PCR prevents false detection of wild-type mtDNA due to heteroduplex formation.

Fluorescence associated cell sorting (FACS)

To assess mitochondrial mass 2.5*10^5 cells were seeded in 6-well plates and allowed to reach 90%–95% confluency. On the day of experiment cells were incubated with normal DMEM containing 50nM MitoTracker Green FM (Thermo Fisher Scientific, cat. no. M7514) for 30 minutes. Cells were detached with 0.25% trypsin and washed three times with PBS. Washed cells were then analyzed by FACS using a LSRII (BD) flow cytometer by monitoring the fluorescence emission at 530 nm ± 15 nm upon excitation with a 488 nm laser. FACS data were analyzed with FlowJo software (Treestar).

NADH measurements

To measure whole cell NAD^+/NADH we used an adapted version of the method proposed by Frezza et al. (2011). 6*10^4 cells were seeded in a 96-well plate the day before experiment and an enzymatic cycling reaction was performed. On the day of experiment cells were washed twice with PBS and 100 μL of EB-DTAB buffer (1% w/v dodecyltrimethylammonium bromide (DTAB), 20 mM sodium bicarbonate, 100 mM sodium carbonate, 10 mM nicotinamide, 0.05% v/v Triton X-100, pH 10.3) was added into each well, cell lysis was facilitated by pipetting and 50 μL of lysed cells were transferred into a new empty well. 25 μL of 0.4 N HCl were added to the last well (acid-treated sample) and plate was incubated at 60°C for 15 minutes. Plate was then equilibrated at room temperature for 10 minutes and 25 μL of 0.5 M Trizma base were added to the acid-treated wells. 50 μL of HCl/Trizma solution (0.4 N HCl: 0.5 M Trizma base 1:1 v/v) were added to the untreated wells (base-treated samples). 5 μL of each acid-treated and base-treated samples were transferred to a new 96-well plate and 195 μL of cycling solution (CS) were added. CS was composed of 84% v/v of reaction cocktail (120 mM bicine, 3.7% EtOH, 5 mM EDTA, pH 7.8), 3% v/v of 5 mg/mL alcohol dehydrogenase (ADH, Sigma-Aldrich cat. no. A3263) in ddH_2O, 8.5% v/v 20 mM Phenazine Thosulfate (PES, Sigma-Aldrich cat. no. P4544), 4.5% v/v 10 mM 3-[4,5-Dimethylthiazole-2-yl]-2,5-diphenyltetrazolium Bromide (MTT, Sigma-Aldrich cat. no. M2128). Plate was incubated at room temperature for 30 minutes and MTT absorbance was read at 570 nm with a Tecan 200 Pro microplate reader. Blank samples composed of EB-DTAB buffer only were prepared and subtracted to all other samples. Standard curves with NAD^+ and NADH in the range of 0.05-5 μM were prepared for quantification.

To assess mitochondrial NADH 5*10^5 the day before experiment cells were seeded on a 15 mm coverslip and incubated overnight in standard medium. The next day medium was washed and replaced with 500 μL of phenol red-free DMEM (Sigma-Aldrich cat. no. D5030) supplemented with 25 mM glucose, 1 mM pyruvate, 2 mM glutamax (Thermo Fisher Scientific cat. no. 35050061), 10 mM HEPES, pH 7.4. Each coverslip was then placed in a metal ring and fitted on a heated stage at 37°C. NAD(P)H fluorescence intensity time series were performed on an inverted LSM 510 laser scanning confocal microscope (Carl Zeiss) with 351nm illumination from an argon ion laser (Coherent Enterprise UV). NAD(P)H fluorescence was detected using a 351-nm long-pass dichroic and 460 ± 25nm band-pass emission filter with a × 40, 1.3 NA quartz oil immersion objective. Images (12-bit 512 × 512) were obtained with a pixel dwell time of 1.6μs. To reduce noise, the image recorded at each time point was an average of two consecutive scans. Time series measurements were obtained by acquiring one image every minute following this pattern: 1) basal conditions (5 minutes); 2) dropwise addition of 100 μL of 6 mM cyanide was added (1 mM final concentration, 4 minutes); 3) replacement of medium with 800 μL of fresh medium (5 minutes); 4) dropwise addition of 200 μL of 5 μM FCCP (1 μM final concentration, 4 minutes). Three coverslips per condition were assayed in each experiment and three independent experiments were carried out. Images were analyzed with ImageJ 1.49. Same value (1280) of thresholding was used to detect objects in each image for each condition, watershed processing was applied and intensity was analyzed by detecting particles larger than 20 pixels. Determination of basal NADH levels in all conditions were obtained by calculating the average intensity value after CN addition and setting this value to 100 (maximal intensity). Intensity values after FCCP addition were set to 0 (minimal intensity). Basal levels of NADH were calculated by referring basal fluorescence levels to the calculated maximal and minimal intensity values.

Metabolomics analysis

For steady-state metabolomics or metabolite tracing experiments 1.2*10^5 cells were seeded in 12-well plates. After 24 hours cells were washed twice with PBS and medium was changed with normal DMEM or medium containing metabolite tracers. For glucose tracing experiments 25 mM U-^{13}C-glucose (Cambridge Isotope Laboratories, cat. no. CLM-1396-MPT-PK) or 4-^{2}H-glucose (Cambridge Isotope Laboratories, cat. no. DLM-9294-PK) was added to glucose-free and pyruvate-free DMEM (Life Technology cat. no. 11966-025), together with 10% v/v FBS and 1 mM sodium pyruvate. For glutamine tracing experiments 4 mM U-^{13}C-glutamine (Cambridge Isotope Laboratories, cat. no. CLM-1822-SP-PK) or 1-^{13}C-glutamine (Cambridge Isotope Laboratories, cat. no. CLM-3612-PK) was added to glutamine-free DMEM (Life Technology cat. no. 21969-0.35), together with 10% v/v FBS. For aspartate labeling experiments 4 mM U-^{13}C-aspartate (Cambridge Isotope Laboratories, cat. no. CLM-1801-H) was added to normal DMEM with 10% v/v FBS. After incubation with normal medium or medium containing metabolite tracers, one well from each condition was used to estimate cell number. To extract extracellular metabolites, 50 μL of medium were collected from each well, centrifuged at 10000 g for 1 minute and metabolites were extracted by adding 750 μL of metabolite extraction buffer (MEB, 50% v/v methanol, 30% v/v

acetonitrile, 20% v/v ddH_2O). To extract intracellular metabolites, cell plates were placed on ice, washed twice with ice-cold PBS and 1mL of MEB / 10^6 cells was added to each well and cells were scraped. One cycle of freeze-thawing at $-80^{\circ}C$ was performed to further lyse the cells. Both extracellular and intracellular fractions were then incubated in a thermomixer (Eppendorf) at max speed for 15 minutes at $4^{\circ}C$. Proteins were then pelleted by centrifuging samples at 16000 g for 10 minutes at $4^{\circ}C$ and supernatants were transferred into glass vials and stored at $-80^{\circ}C$ until further analysis.

Liquid chromatography–mass spectrometry (LCMS) analysis was performed on a QExactive Orbitrap mass spectrometer coupled to a Dionex UltiMate 3000 Rapid Separation LC system (Thermo). The LC system was fitted with a SeQuant ZIC-pHILIC (150mm × 2.1mm, 5μm) with the corresponding guard column (20m × 2.1mm, 5μm) both from Merck. The mobile phase was composed of 20cmM ammonium carbonate and 0.1% ammonium hydroxide in water (solvent A), and acetonitrile (solvent B). The flow rate was set at 200 μL/min with a previously described gradient (Mackay et al., 2015). The mass spectrometer was operated in full MS and polarity switching mode scanning a range of 50-750 m/z. Samples were randomized, in order to avoid machine drift, and were blinded to the operator. The acquired spectra were analyzed using XCalibur Qual Browser and XCalibur Quan Browser software (Thermo Scientific) by referencing to an internal library of compounds. Calibration curves were generated using synthetic standards of the indicated metabolites.

Intensity of intracellular metabolites were normalized on total ion sum (normalized intensity values). For interpretation of labeling patterns normalized intensities of isotopologues were further normalized on total isotopologue sum for each metabolite species (proportion of total pool values). Values of consumption and release of extracellular metabolites were normalized on cell counts and values of metabolites measured in fresh medium were subtracted.

Metabolic modeling

Modeling of metabolic rewiring following mitochondrial dysfunction was performed with flux balance analysis (FBA) (Orth et al., 2010), under the assumption of mass conservation. Alternative reaction flux solutions were investigated with flux variability analysis (FVA) (Mahadevan and Schilling, 2003). To investigate metabolic rewiring, we constrained a recently published metabolic reconstruction of central carbon metabolism (Zieliński et al., 2016) with experimental data obtained from consumption and release of extracellular metabolites and oxygen consumption driven by individual complexes (Table S1). We maximized ATP yield as objective function and calculated the flux difference between mT7 and mT80 models by subtracting the predicted flux of each reaction for mT7 from mT80 model. Top 10% altered reactions were considered. To assess contribution of each reaction to ATP production, we individually blocked (upper bound = 0, lower bound = 0) each reaction in mT80 and mT7 models and calculated the difference in ATP yield against the complete model. Contribution of reductive carboxylation to metabolic rewiring was predicted by blocking IDH1 reaction in mT80 model and calculating the flux difference of each reaction against the complete mT80 model. Top 10% altered reactions were considered. Simulations were performed with MATLAB R2016A (MathWorks) with the COBRA toolbox 2.0 and by using GLPK 4.48 as solver.

Lentiviral vectors generation and transduction

The viral supernatant for cell transduction was obtained from the filtered growth media of the packaging cells HEK293T transfected with with 3 μg psPAX, 1 μg pVSVG, 4 μg of shRNA constructs and 24 μl Lipofectamine 2000 (Life Technology). 1 × 10^6 cells were then plated on a 6 cm dish and infected with the viral supernatant in the presence of 4 μg/ml polybrene. After 2 days, the medium was replaced with selection medium containing 2 μg/ml puromycin. The expression of the shRNA constructs was induced by incubating cells with 2 μg/ml doxycycline. The shRNA sequences targeting IDH1, IDH2 or MDH1 were purchased from Thermo Scientific and are as follows: shNTC #RHS4743; shIDH1 #1: TTTCGTATGGTGCCATTTG; shIDH1 #2: TTGACGCCAACATTATGCT; shIDH2 #1: TCTTGGTGCTCATGTACAG; shIDH2 #2: TTCTTGTCGAAGTCGGTCT; shMDH1 #1: CAATTTGAGCTTTAGCTCG; shMDH1 #2: TATTCTTGATTACAACAGG. For NDI-1 expression, 3*10^6 HEK293T cells were plated in 10 cm dishes, allowed to attach overnight, and transfected with 5 μg psPAX, 5 μg pMD2G and 2 μg of pWPI control or NDI-1 plasmids (Cannino et al., 2012). The shRNA sequences targeting GOT1 were kindly donated by the Lyssiotis lab (Son et al., 2013). Viral supernatant was used as described above for infecting mT7 or mT80 cells.

Cell migration

6*10^6 cells were seeded in a 96-well plate and cultured overnight in standard conditions. A 700-800 μm wound was obtained in each well with a 96 pins IncuCyte WoundMaker (Essen Bioscience). After applying the wound, cells were washed with PBS twice and medium was replaced with 100 μL DMEM. Images were acquired with an IncuCyte FLR (Essen Bioscience) every 2 hours for at least 10 consecutive hours. Wound widths at time point 0 hours and 6 hours were extracted and used to calculate migration speed. 8-16 wells per condition were used as technical replicates in each experiment, and at least 4 independent experiments were acquired.

qPCR

mRNA was extracted using RNeasy Kit (QIAGEN) following manufacturer's instructions. 1 μg of mRNA was retrotranscribed into cDNA using High Capacity RNA-to-cDNA Kit (Applied Biosystems, Life Technologies, Paisley, UK). For the qPCR reactions 0.5 μM primers were used. 1 μl of Fast Sybr green gene expression master mix; 1 μl of each primers and 4 μl of 1:10 dilution of cDNA in a final volume of 20 μl were used. Real-time PCR was performed in the Step One Real-Time PCR System (Life Technologies

Corporation Carlsbad, California) using the fast Sybr green program and expression levels of the indicated genes were calculated using the ΔΔCt method by the appropriate function of the software using actin as calibrator. Primer sequences are as follows: IDH1: Fwd: GTGTGCAAAATCTTCAATTGACTT; RV: GGTGACATACCTGGTACATAACTTTG; IDH2: Fwd: GGAGCCCGAGGTCA AAATAC; RV: TGGCAGTTCATCAAGGAGAA; Actin: QuantiTect primer QT00095431 (QIAGEN), sequence not disclosed.

QUANTIFICATION AND STATISTICAL ANALYSIS

Statistical analysis was performed with Graphpad Prism 5.0a. At least 3 independent experiments were used for each test. Statistical analysis of metabolomics data was performed with the R package muma v1.4 (Gaude et al., 2013) on R software 3.3.2. Statistical details of each experiment including statistical test used, number of replicates, center and dispersion measures can be found within corresponding figure legends. False discovery rate (FDR) of 5% was used to determine statistical significance. For metabolomics data analysis multiple hypothesis correction with Benjamini-Hochberg method was applied. For proteomics data analysis differential analysis was performed on the proteomic data using the limma R package (Ritchie et al., 2015). The considered contrast were mT45 against mT7, mT80 against mT7 and mT80 against mT45. An enrichment analysis was performed with the resulting statistics of each considered contrast using the piano R package (Väremo et al., 2013). The gene set collections used were obtained from mSIGDB (c5.GO.bp). Each gene set collection was enriched independently. The consensus enrichment p value for each considered gene set was obtained using all available methods of the piano package, except GSEA. The p values were corrected with the Benjamini-Hochberg procedure for false discovery rate.

DATA AND SOFTWARE AVAILABILITY

Metabolites detected in our metabolomics analysis, together with corrected ANOVA p values are reported in Table S1. Consumption and release (CORE) data used to constrain the metabolic model is reported in Table S2. Flux range predictions obtained with FVA are reported in Table S3. GSEA data obtained from proteomics analysis of mT7 and mT80 cells are reported in Table S4. Raw images have been deposited to Mendeley Data and are available at http://dx.doi.org/10.17632/bmvrzxgs6c.1.

Molecular Cell, Volume *69*

Supplemental Information

NADH Shuttling Couples Cytosolic Reductive Carboxylation of Glutamine with Glycolysis in Cells with Mitochondrial Dysfunction

Edoardo Gaude, Christina Schmidt, Payam A. Gammage, Aurelien Dugourd, Thomas Blacker, Sew Peak Chew, Julio Saez-Rodriguez, John S. O'Neill, Gyorgy Szabadkai, Michal Minczuk, and Christian Frezza

Supplementary Figure 1

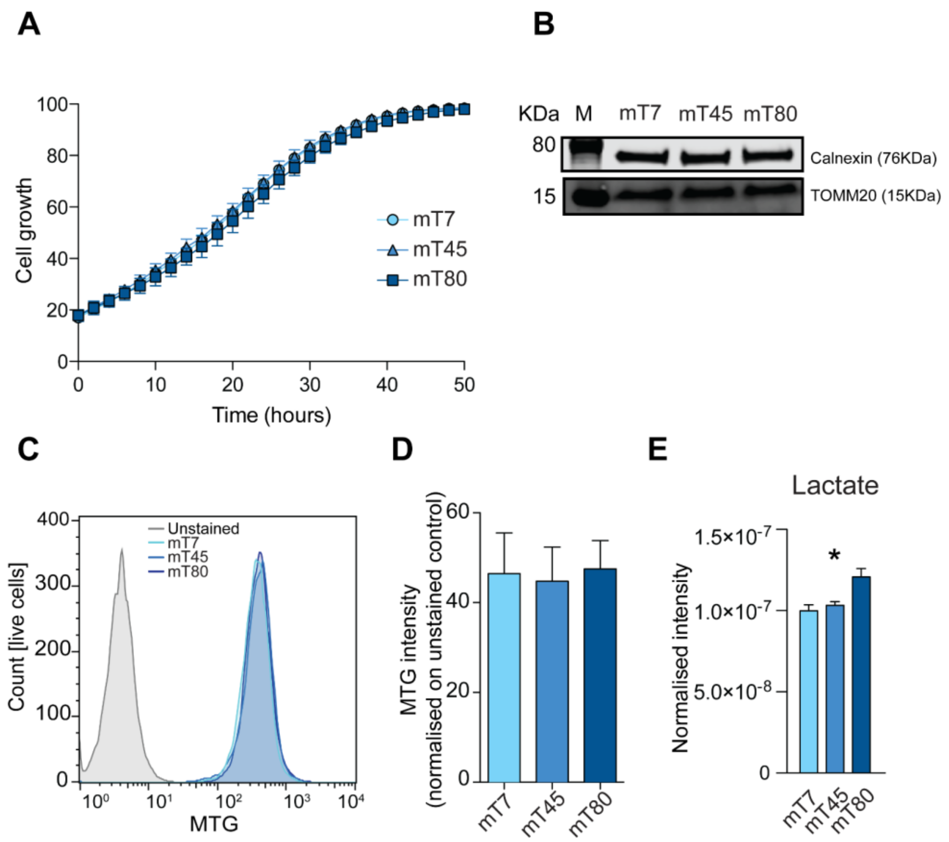

Supplementary Figure 1 related to Figure 1. Characterisation of cybrid cells. (A) Cell growth of mT7, mT45, and mT80 cells in standard conditions measured using Incucyte. (B) Western blot analysis of the mitochondrial membrane marker TOMM20. Calnexin was used as loading control. (C) Representative fluorescence distributions of cells with or without Mitotracker Green (MTG) staining from FACS analysis and (D) mean MTG intensity after subtraction of intensity from unstained control. Data are mean ± s.e.m. from three independent cultures. (E) Lactate secretion as measured in mT7, mT45 and mT80. All data are mean ± s.e.m. from four independent cultures. (E) * indicates ANOVA p-value < 0.05, respectively.

Supplementary Figure 2

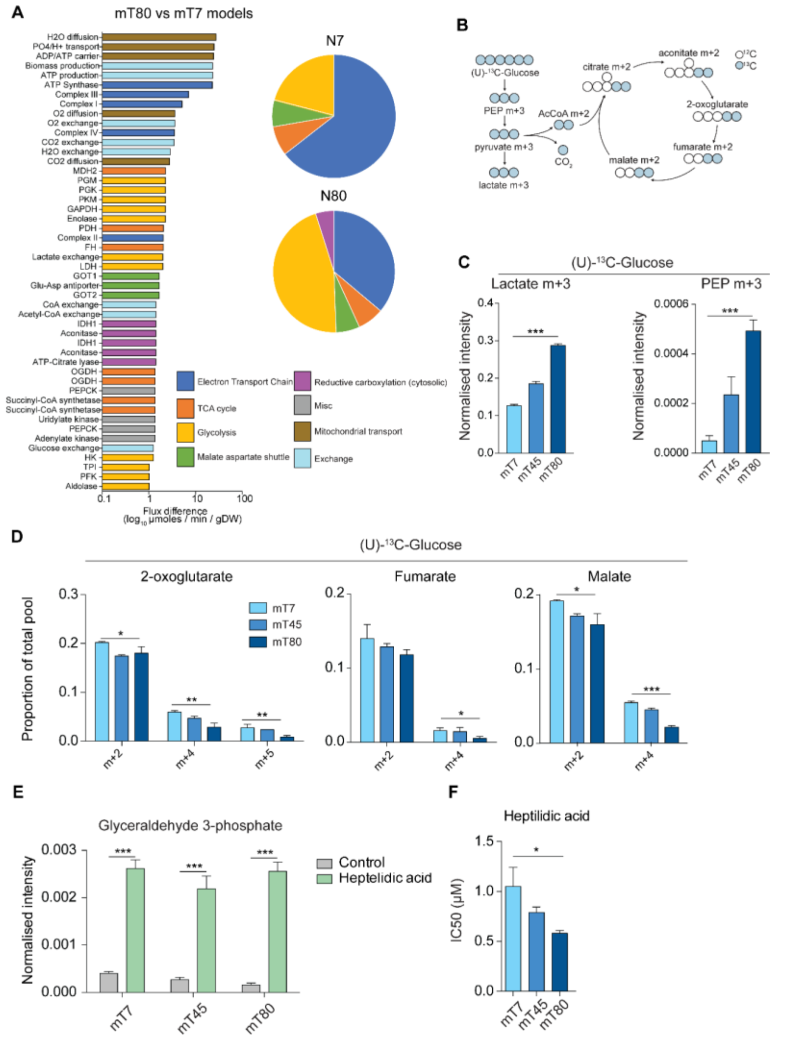

Supplementary Figure 2 related to Figure 2. Metabolic features of mTUNE cells. (A) Top 10% affected reactions between mT80 and mT7 as predicted by metabolic modelling. Flux difference between mT80 and mT7 model is shown in $\log_{10}$ scale. Each reaction is colour-coded based on distribution into metabolic pathways. Duplicated reactions indicate intermediate of reactions. (B) Schematic representation of metabolite labelling pattern from (U)-^{13}C-glucose. (C) Normalised intensities (total metabolite count) of lactate m+3 (left) and phosphoenolpyruvate (PEP) m+3 (right) after incubation with (U)-^{13}C-glucose. (D) Labelling patterns of the indicated TCA cycle intermediates after incubation with (U)-^{13}C-glucose. (E) Normalised intensity (total metabolite count) of glyceraldehyde 3-phosphate (GA3P) upon treatment of mT7, mT45 and mT80 cells with vehicle control or 0.5 μM of the GAPDH inhibitor heptelidic acid. Data are mean ± s.e.m. from 3 independent cultures. (F) IC50 values of heptelidic acid on the proliferation of mT7, mT45 and mT80 cells. All data are mean ± s.e.m. from at least three independent cultures. (C-D, F) *, ** and *** indicate one-way ANOVA p-value ≤ 0.05, 0.001 and 0.001, respectively. (E) *** indicates two-sided t-test p-value ≤ 0.001.

Supplementary Figure 3

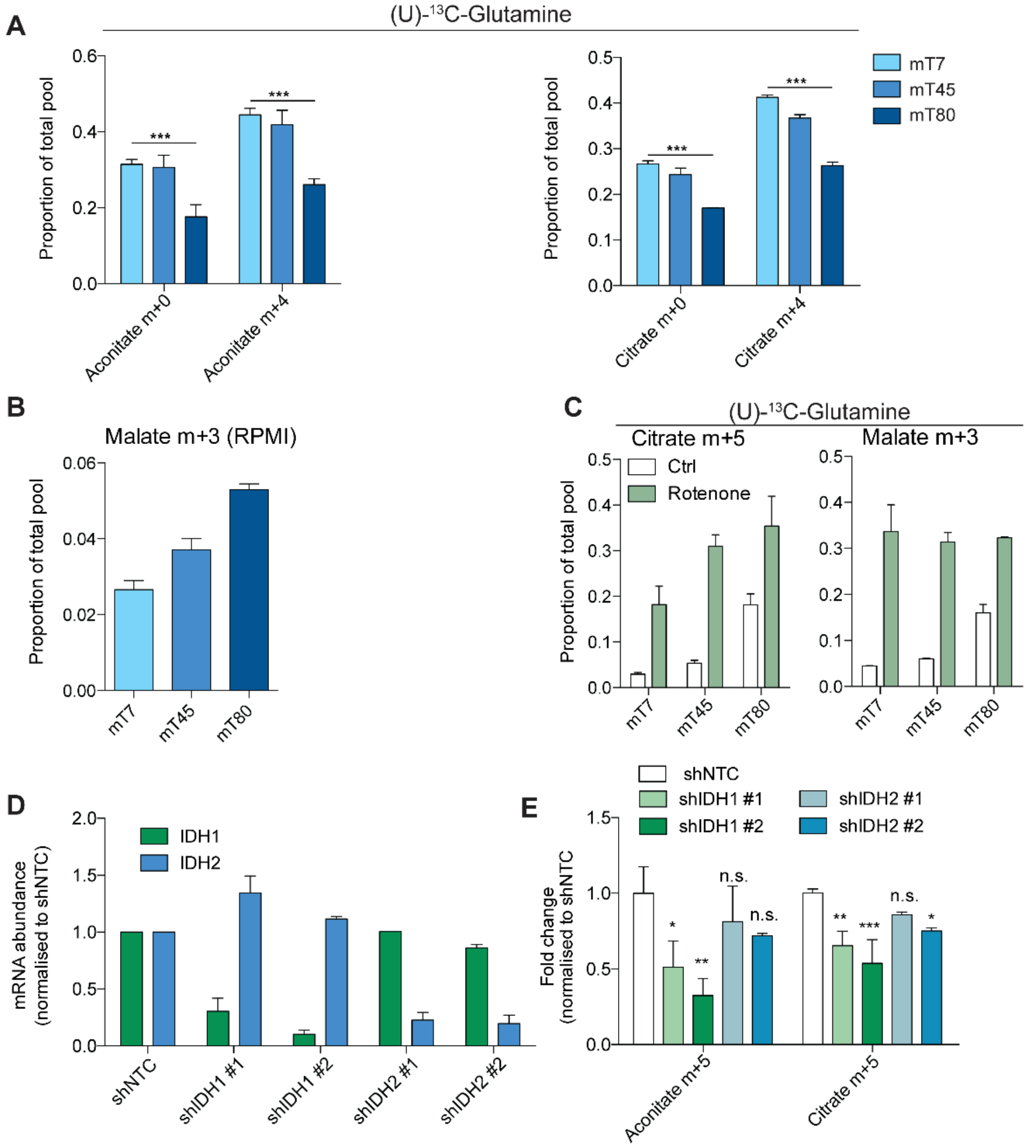

Supplementary Figure 3 related to Figure 2. Assessment of reductive carboxylation in the cybrid cells. (A) Labelling patterns of the indicated TCA cycle intermediates after incubation with U-^{13}C-glutamine. (B) Proportion of total pool of malate m+3 originating from U-^{13}C-glutamine in mT7, mT45 and mT80 cells grown in RPMI medium. Data are mean ± s.d. from one representative experiment. (C) Proportion of total pool of citrate m+5 and malate m+3 originating from U-^{13}C-glutamine in mT7, mT45 and mT80 cells in the presence of 0.5 µM rotenone. Data are mean ± s.d. from one representative experiment. (D) mRNA expression of IDH1 and IDH2 genes upon selective knock down of IDH1 or IDH2 with two independent shRNA constructs. Actin mRNA expression was used as endogenous control and data were normalised on shNTC control. Data are mean ± s.d. from one representative experiment. (E) Levels of metabolites from reductive carboxylation of U-^{13}C-Glutamine in mT80 cells infected with shRNA constructs targeting IDH1 or IDH2. Data are normalised on non-targeting shRNA control (shNTC). (A, E) Data are mean ± s.e.m. from three independent cultures. (A) *** indicates one-way ANOVA p-value ≤ 0.001. (C) *, **, *** indicate Dunnett's p-value ≤ 0.05, 0.01 and 0.001, respectively. n.s. = not significant.

Supplementary Figure 4

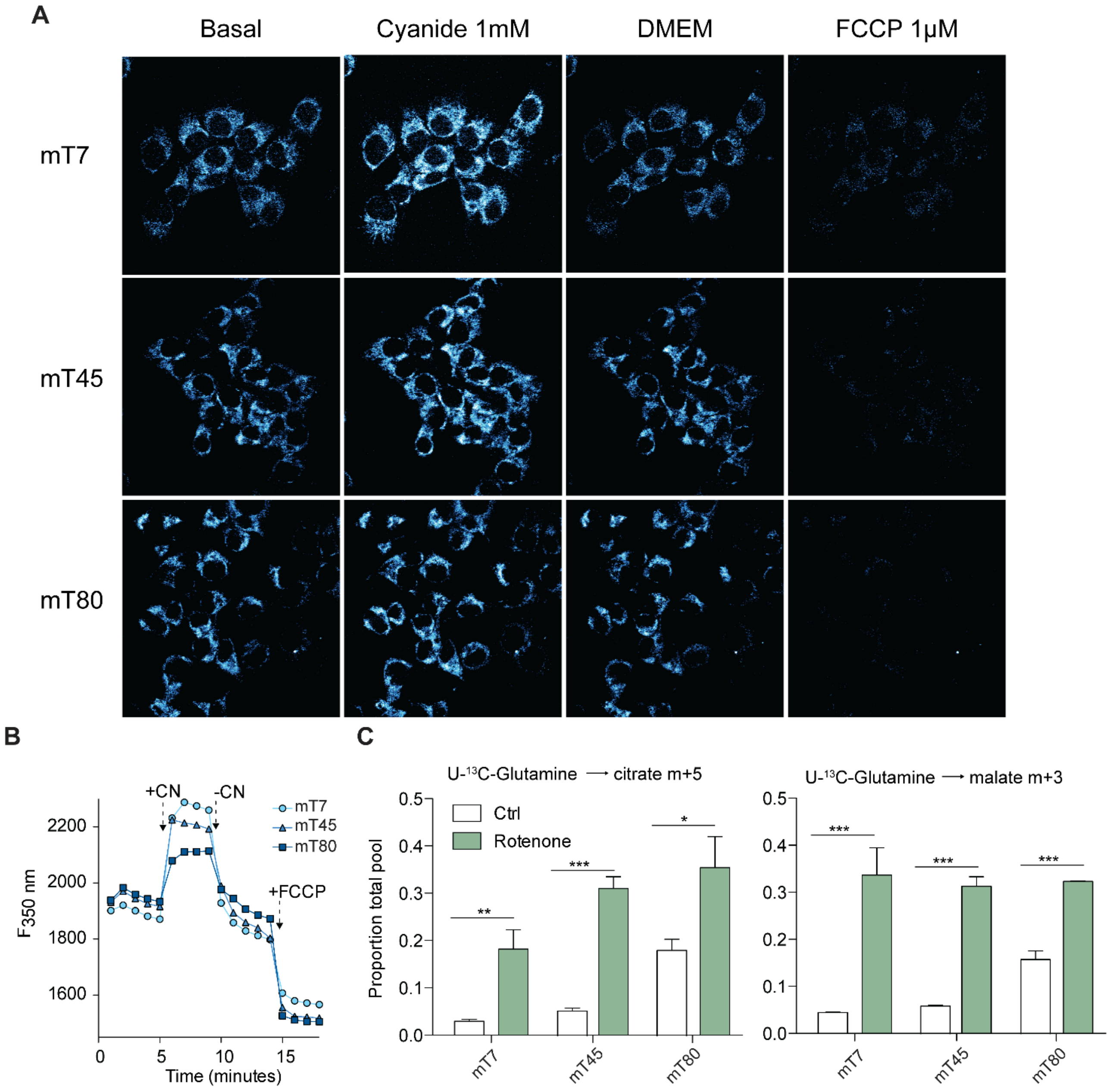

Supplementary Figure 4 related to Figure 3. Mitochondrial NADH in the cybrid cells. (A) Representative images of mT7, mT45 and mT80 cells excited at 350 nm. (B) Fluorescence intensity after excitation at 350 nm extracted from image time series analysis of mT7, mT45 and mT80 cells in basal conditions or after addition of 1 mM cyanide (CN), medium replenishment and addition of 1 μM FCCP. (C) Proportion of total pool for citrate m+5 and malate m+3 in mT7, mT45, and mT80 cells treated with vehicle control or 0.5 μM rotenone. Data are mean ± s.e.m. from three independent cultures. *, ** and *** indicate one-way ANOVA p-value ≤ 0.05, 0.001 and 0.001, respectively.

Supplementary Figure 5

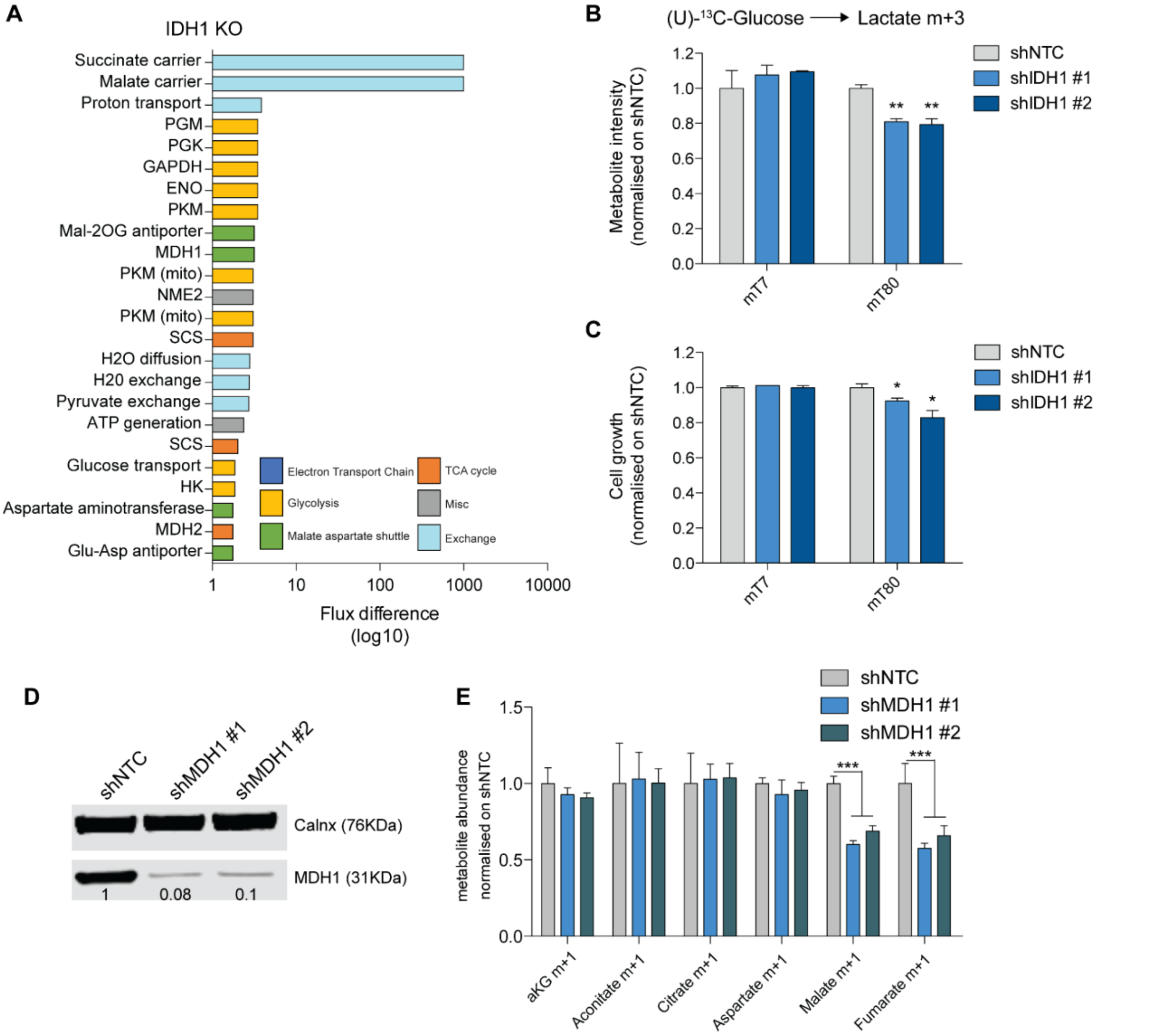

Supplementary Figure 5 related to Figure 4. The role of reductive carboxylation in the cybrid cells. (A) Top 10% affected reactions between mT80 and mT7 as predicted by metabolic modelling. Each reaction is colour-coded based on distribution into metabolic pathways. (B) Levels of lactate secretion in mT7 and mT80 cells upon silencing of IDH1. Data are normalised on shNTC controls and are ± s.e.m. from three independent cultures. (C) Cell growth of mT7 and mT80 cells upon silencing of IDH1. Data are normalised on shNTC controls and are ± s.e.m. from three independent cultures. (D) Western blot analysis of MDH1 expression upon infection of mT80 cells with non-targeting control (shNTC) or shRNA constructs targeting MDH1 (shMDH1 #1 and #2). Densitometry values are shown. Representative blot of three independent experiments. (E) Levels of metabolites from 1-^{13}C-Glutamine in shMDH1 mT80 cells. Data are normalised on non-targeting shRNA control (shNTC). *, **, ** indicates two-sided t-test p-value ≤ 0.05, 0.01 and 0.001,

Supplementary Figure 6

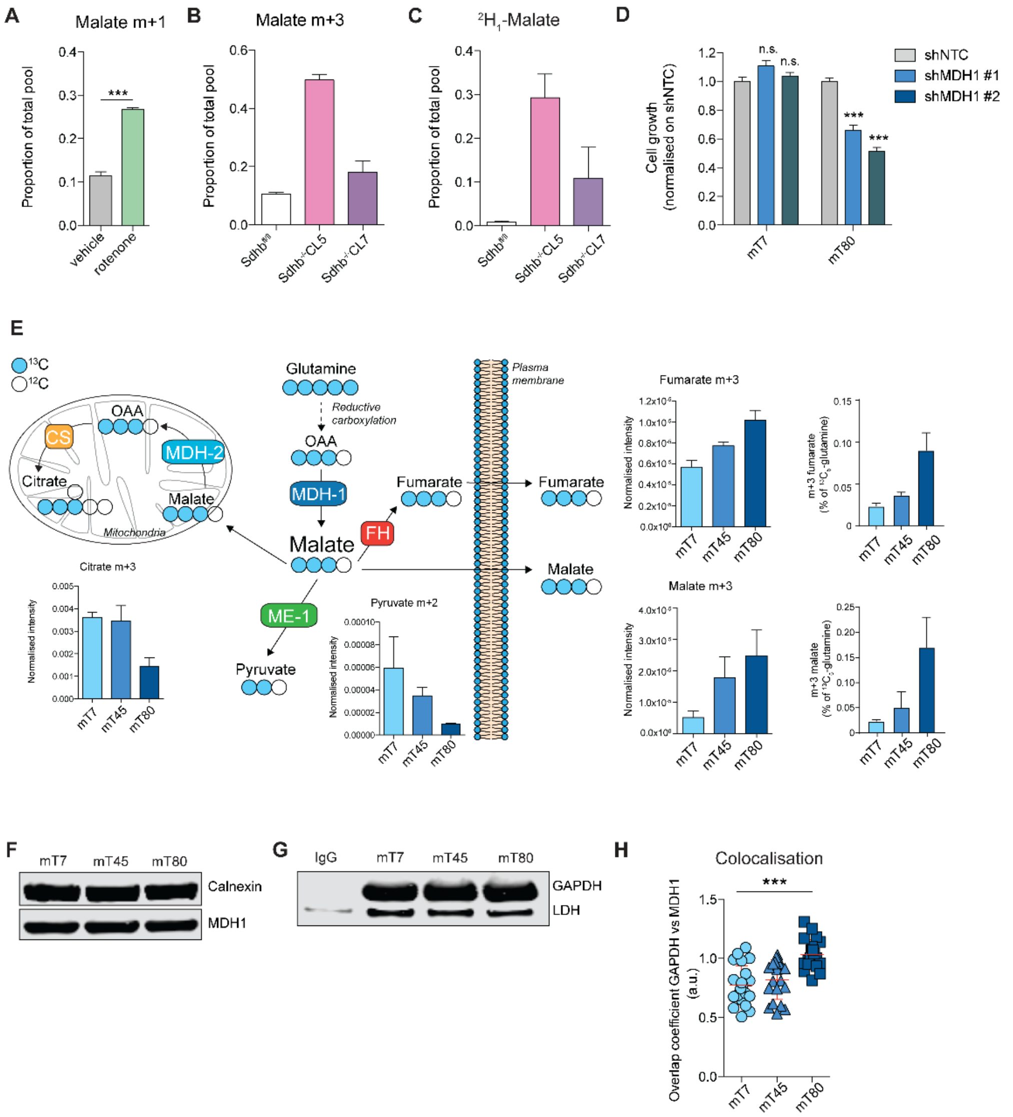

Supplementary Figure 6 related to Figure 4. Functional interaction between MDH1 and GAPDH. (A) Proportion of total pool for malate m+1 in mT7 cells treated with vehicle control or 0.5 μM rotenone and incubated with 1-^{13}C-glutamine. (B-C) Proportion of total pool of malate m+3 (B) and ^{2}H-malate in SDH null cells grown in the presence of (U)-^{13}C-glutamine (B) or 4-^{2}H-glucose (C). Data are mean ± s.d. from one representative experiment. (D) Cell growth of mT7 and mT80 cells upon silencing of MDH1. Data are normalised on shNTC controls and are ± s.e.m. from three independent cultures. (E) Intracellular levels of citrate m+3 and pyruvate m+2, and extracellular levels of malate m+3 and fumarate m+3 in mTUNE cells grown in the presence of (U)-^{13}C-glutamine. The percentage of m+3 fumarate and malate produced vs glutamine consumed (moles released/consumed in 24 hours), are indicated. Data are mean ± s.d. from one representative experiment. (F) Western blot analysis of MDH1 expression in mT7, mT45, and mT80 cells. Calnexin was used as loading control. (G) Immunoprecipitation analysis of GAPDH in mT7, mT45 and mT80 cells. Mouse IgG were used as isotype control for the IP reaction. (H) Co-localisation of fluorescence signals from GAPDH and MDH1 as detected in mT7, mT45, and mT80 by immunofluorescence. Data are mean ± s.d. from 20-30 ROIs per conditions. (A, D) *** indicates two-sided t-test p-value ≤ 0.001. (H) *** indicates one-way ANOVA p-value ≤ 0.001.

Supplementary Figure 7

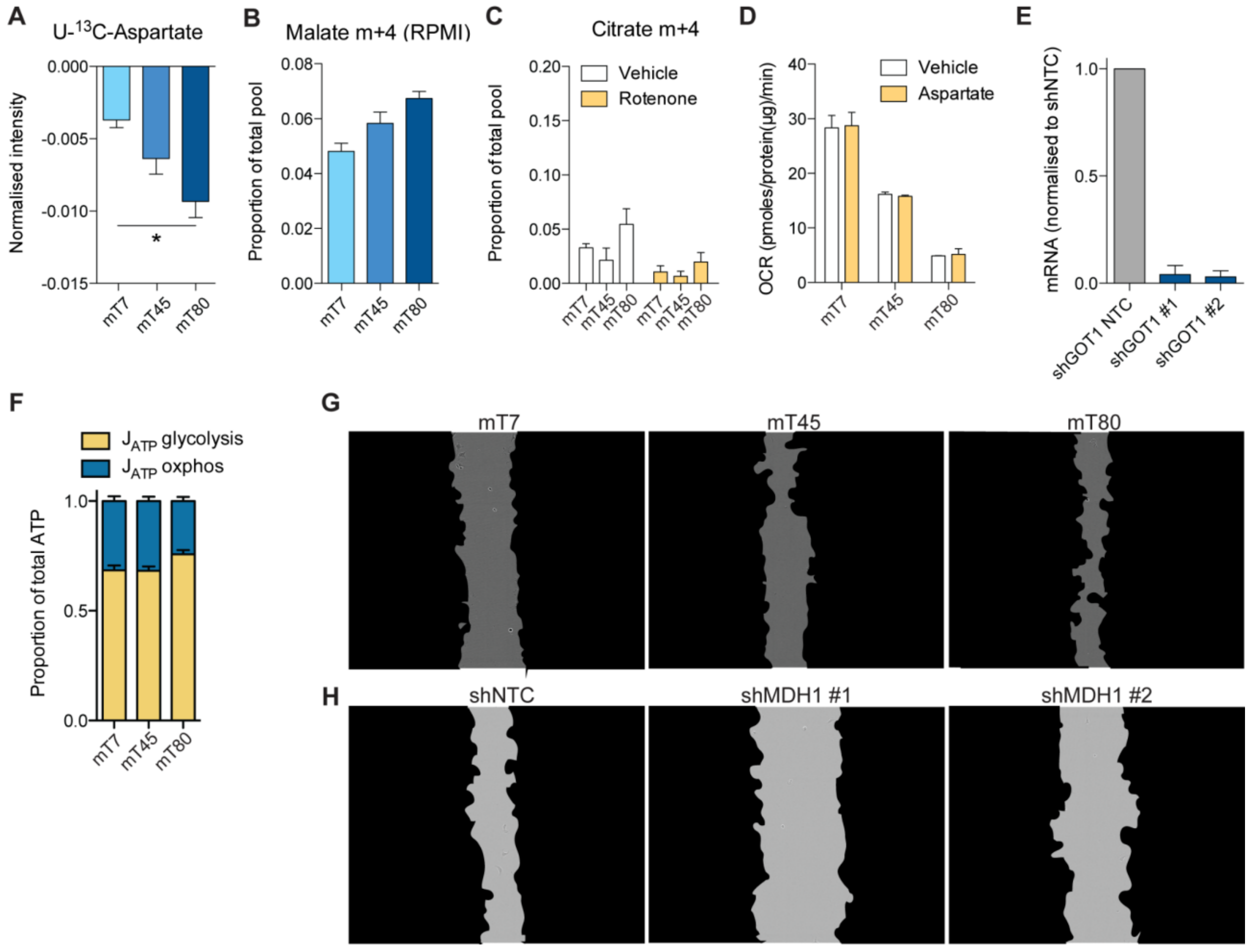

Supplementary Figure 7 related to Figure 5. Aspartate metabolism in the cybrid cells. (A) Consumption of (U)-^{13}C-aspartate from extracellular medium in mTUNE cells. * indicates one-way ANOVA p-value ≤ 0.05. (B) Proportion of total pool of malate m+4 upon supplementation with (U)-^{13}C-aspartate in mTUNE cells grown in RPMI medium. (C) Proportion of total pool for citrate m+4 in mT7, mT45 and mT80 cells treated with vehicle control or 0.5 µM rotenone and incubated with U-13C-aspartate. (D) Oxygen consumption rate (OCR) of mT7, mT45, and mT80 cells upon addition of vehicle control or 5 mM aspartate. (E) mRNA levels of GOT1 in mT80 shGOT1 cells. Data are mean ± s.d. from one representative experiment. (F) Proportion of total ATP originating from glycolysis and oxidative phosphorylation in mT7, mT45, and mT80 cells, as calculated from OCR and ECAR measurements. (G-H) Representative images of wound-healing assay 6 hours after application of wound in mT7, mT45, and mT80 (G) and shMDH1 (H) cells.
